# Highly Purified Alloantigen-Specific Tregs From Healthy and Chronic Kidney Disease Patients Can Be Long-Term Expanded, Maintaining a Suppressive Phenotype and Function in the Presence of Inflammatory Cytokines

**DOI:** 10.3389/fimmu.2021.686530

**Published:** 2021-10-28

**Authors:** Arimelek Cortés-Hernández, Evelyn Katy Alvarez-Salazar, Saúl Arteaga-Cruz, Katya Rosas-Cortina, Nadyeli Linares, Josefina M. Alberú Gómez, Gloria Soldevila

**Affiliations:** ^1^ Department of Immunology, Instituto de Investigaciones Biomédicas, Universidad Nacional Autónoma de México, Mexico City, Mexico; ^2^ National Laboratory of Flow Cytometry, Instituto de Investigaciones Biomedicas, Universidad Nacional Autónoma de México, Mexico City, Mexico

**Keywords:** transplantation, regulatory T cells, allospecific, expansion, suppression

## Abstract

The adoptive transfer of alloantigen-specific regulatory T cells (^allo^Tregs) has been proposed as a therapeutic alternative in kidney transplant recipients to the use of lifelong immunosuppressive drugs that cause serious side effects. However, the clinical application of ^allo^Tregs has been limited due to their low frequency in peripheral blood and the scarce development of efficient protocols to ensure their purity, expansion, and stability. Here, we describe a new experimental protocol that allows the long-term expansion of highly purified allospecific natural Tregs (nTregs) from both healthy controls and chronic kidney disease (CKD) patients, which maintain their phenotype and suppressive function under inflammatory conditions. Firstly, we co-cultured CellTrace Violet (CTV)-labeled Tregs from CKD patients or healthy individuals with allogeneic monocyte-derived dendritic cells in the presence of interleukin 2 (IL-2) and retinoic acid. Then, proliferating CD4^+^CD25^hi^CTV^−^ Tregs (allospecific) were sorted by fluorescence-activated cell sorting (FACS) and polyclonally expanded with anti-CD3/CD28-coated beads in the presence of transforming growth factor beta (TGF-β), IL-2, and rapamycin. After 4 weeks, ^allo^Tregs were expanded up to 2,300 times the initial numbers with a purity of >95% (CD4^+^CD25^hi^FOXP3^+^). The resulting allospecific Tregs showed high expressions of CTLA-4, LAG-3, and CD39, indicative of a highly suppressive phenotype. Accordingly, expanded ^allo^Tregs efficiently suppressed T-cell proliferation in an antigen-specific manner, even in the presence of inflammatory cytokines (IFN-γ, IL-4, IL-6, or TNF-α). Unexpectedly, the long-term expansion resulted in an increased methylation of the specific demethylated region of *Foxp3*. Interestingly, ^allo^Tregs from both normal individuals and CKD patients maintained their immunosuppressive phenotype and function after being expanded for two additional weeks under an inflammatory microenvironment. Finally, phenotypic and functional evaluation of cryopreserved ^allo^Tregs demonstrated the feasibility of long-term storage and supports the potential use of this cellular product for personalized Treg therapy in transplanted patients.

## Introduction

Kidney transplantation is currently the therapy of choice for most causes of chronic kidney disease (CKD) (1). To prevent allograft rejection, kidney transplant (KT) patients are treated with immunosuppressive drugs that reduce the rates of renal acute rejection and increase patients’ survival (2). However, the long-term use of immunosuppressants can have adverse side effects in KT patients, such as the increase of neoplasias, infections, and cardiovascular diseases, which in turn represent some of the main causes of death in these patients (3). Therefore, it is still necessary to develop new therapies that induce specific allograft tolerance.

Regulatory T cells (Tregs) have a crucial role in establishing and maintaining peripheral immune tolerance. Tregs are characterized by the expression of FOXP3, a transcription factor that regulates their immunosuppressive function (4). Studies in mouse models have demonstrated that Tregs are essential to inducing specific KT tolerance by the inhibition of effector T cells and modulating dendritic cell function (5). KT patients who developed clinical operational tolerance exhibit a significant increase in the number of circulating FOXP3^+^ Tregs (6, 7). Hence, the clinical application of Tregs has been extensively studied as an approach for the induction of allotransplant tolerance (8).

Due to the low frequency of Tregs in peripheral blood (<1% of white blood cells), several protocols for *ex vivo* expansion of Tregs have been designed to obtain the cell numbers required for immunotherapy (8). Following this approach, clinical trials using polyclonal Tregs have been implemented in humans, with hematopoietic stem cell transplantation (HSCT) reporting a significant decrease of the severity of graft *versus* host disease (GvHD) (9, 10). Similarly, phase I or phase I/IIa studies adopting expanded Tregs have recently been performed in KT patients (11–13), which reported neither infusion-related side effects nor increased infections or rejection events during post-transplant, evidencing the safety of Treg therapy. Nonetheless, these studies have used polyclonal Tregs, and preclinical studies have indicated that adoptive transfer of alloantigen-specific Tregs (^allo^Tregs) may be a better approach to inducing long-term allograft acceptance (14–17).

The high precursor frequency of natural Tregs (nTregs) recognizing alloantigens directly (5%–10% of blood Tregs) compared with indirectly (<0.1%) (18) has promoted the development of Treg expansion protocols based on direct allorecognition (19). However, the large-scale production of human allospecific Tregs for immunotherapy has remained a challenge due to the lack of optimized protocols to allow their purification and efficient expansion, preserving their functional and phenotypic stability (19). In addition, studies have shown that long-term expansion of Tregs results in the loss of FOXP3 and may convert to potentially inflammatory T cells (20, 21). Recently, our group has applied a protocol that allows the generation of a large number of functionally stable allogeneic induced Tregs (iTregs) after long-term polyclonal expansion (22). Finally, another important issue recently addressed to optimize the function of infused Tregs for the induction of effective tolerance toward the allograft is the homing capabilities of the infused Tregs (23).

In the present study, we describe a new protocol where the increased expansion and survival of long-term stimulated ^allo^Tregs allows the production of highly purified allospecific Tregs from healthy individuals and patients with CKD that maintain a suppressive phenotype and suppressor function in the presence of pro-inflammatory cytokines, supporting the potential of *in vitro* expanded allospecific Tregs for immunotherapy in kidney transplantation.

## Materials and Methods

### Patients With Chronic Kidney Disease

The present study was approved by the Committees of Medical Ethics and Research at the Instituto de Investigaciones Biomédicas (UNAM) and the Instituto Nacional de Ciencias Médicas y Nutrición Salvador Zubirán (reference #1831) and was performed in accordance with the revised Declaration of Helsinki, the Declaration of Istanbul, and Good Clinical Practice Guidelines. All patients provided written informed consent to participate in the study and were maintained in renal replacement therapy while awaiting kidney transplant.

Buffy coat preparations of blood from healthy individuals (control group) were provided by the Blood Bank of the Instituto Nacional de Enfermedades Respiratorias, México.

### Reagents and Antibodies

For flow cytometry, allophycocyanin (APC) anti-CD4, PerCP-Cy5.5 anti-CD4, PE-Cy7 anti-CD8, APC anti-CD11c, phycoerythrin (PE) anti-CD86, fluorescein isothiocyanate (FITC) anti-CD14, and Foxp3/Transcription Factor Staining Buffer Kit were obtained from Tonbo Biosciences (San Diego, CA, USA). Alexa Fluor 647 anti-FOXP3 was from Beckman Coulter (Brea, CA, USA). PE-Cy5.5 anti-CD3 was from Invitrogen (Waltham, MA, USA). APC-Cy7 anti-human leukocyte antigen DR isotype (HLA-DR), Brilliant Violet 711 anti-CD39, Brilliant Violet 421 anti-CTLA-4, PE anti-CD25, PE-Cy7 anti-CD127, PE-Cy7 anti-LAG-3, FITC anti-Helios, and Zombie Aqua™ were purchased from Biolegend (San Diego, CA, USA).

For *in vitro* experiments, rapamycin, retinoic acid, Ficoll^®^ Paque Plus, and dimethyl sulfoxide (DMSO) were obtained from Sigma-Aldrich (San Louis, MO USA). Recombinant human granulocyte–macrophage colony-stimulating factor (GM-CSF), interferon gamma (IFN-γ), interleukin 2 (IL-2), IL-4, IL-6, transforming growth factor beta (TGF-β), and tumor necrosis factor alpha (TNF-α) cytokines were from PeproTech (Rocky Hill, NJ, USA). Carboxy fluorescein succinimidyl ester (CFSE), CellTrace Violet (CTV), Dynabeads Human T-Activator CD3/CD28 (anti-CD3/anti-CD28-coated beads), DynaMag-5™ Magnet (DynaMag), CTS™ OpTmizer™ T Cell Expansion SFM medium (expansion medium), RPMI 1640 medium, antibiotic–antimycotic 100×, l-glutamine (GlutaMAX™), sodium pyruvate (100 mM), Minimum Essential Medium non-essential amino acids (MEM-NEAA, 100×), and fetal bovine serum (FBS) were obtained from Thermo Fisher Scientific (Waltham, MA, USA). Pooled human AB serum was obtained from Gemini Bio Products (Sacramento, CA, USA). All culture media were supplemented with l-glutamine, sodium pyruvate, MEM-NEAA, and antibiotic-antimycotic. The cultures of T cells were performed in round bottom 96-well culture plates (Corning, Avon, France).

### Isolation and Cryopreservation of PBMCs

Peripheral blood mononuclear cells (PBMCs) were isolated from blood of healthy individuals, patients with CKD, and their potential living kidney donors by density gradient centrifugation over Ficoll^®^ according to the manufacturer’s instructions. A portion of PBMCs was resuspended in a cold freezing medium (10% DMSO and 90% FBS) at a concentration of 10^6^ cells/ml, stored for 24 h at −70°C, and then transferred to liquid nitrogen. For functional assays, the cells were thawed in a 37°C water bath, washed twice with RPMI medium supplemented with 10% FBS, and resuspended in culture medium.

### Monocyte-Derived Dendritic Cells

CD14^+^ monocytes were purified from PBMCs using the Human CD14 MicroBeads Kit (Miltenyi Biotec, Bergisch Gladbach, Germany) according to the manufacturer’s instructions. Isolated CD14^+^ monocytes from kidney donors or healthy controls were cultured in RPMI medium supplemented with 10% human AB serum and stimulated with IL-4 (50 ng/ml) and GM-CSF (50 ng/ml) for 8 days. On days 3 and 5, the culture medium and cytokines (25 ng/ml of IL-4 and GM-CSF) were refreshed. On day 8, monocyte-derived dendritic cells (Mo-DCs) were washed twice with the culture medium and irradiated 3000 rad before the functional assays. A proportion of Mo-DCs was stained with anti-CD14, anti-CD86, anti-CD11c, anti-HLA-DR, and Zombie Aqua™. Then, the cells were acquired on the Attune NxT Flow Cytometer (Thermo Fisher Scientific) and the data analyzed with FlowJo vX.0.7 software (Tree Star, Covington, KE, USA).

### Isolation and Expansion of Allospecific Tregs

**Figure 1:**
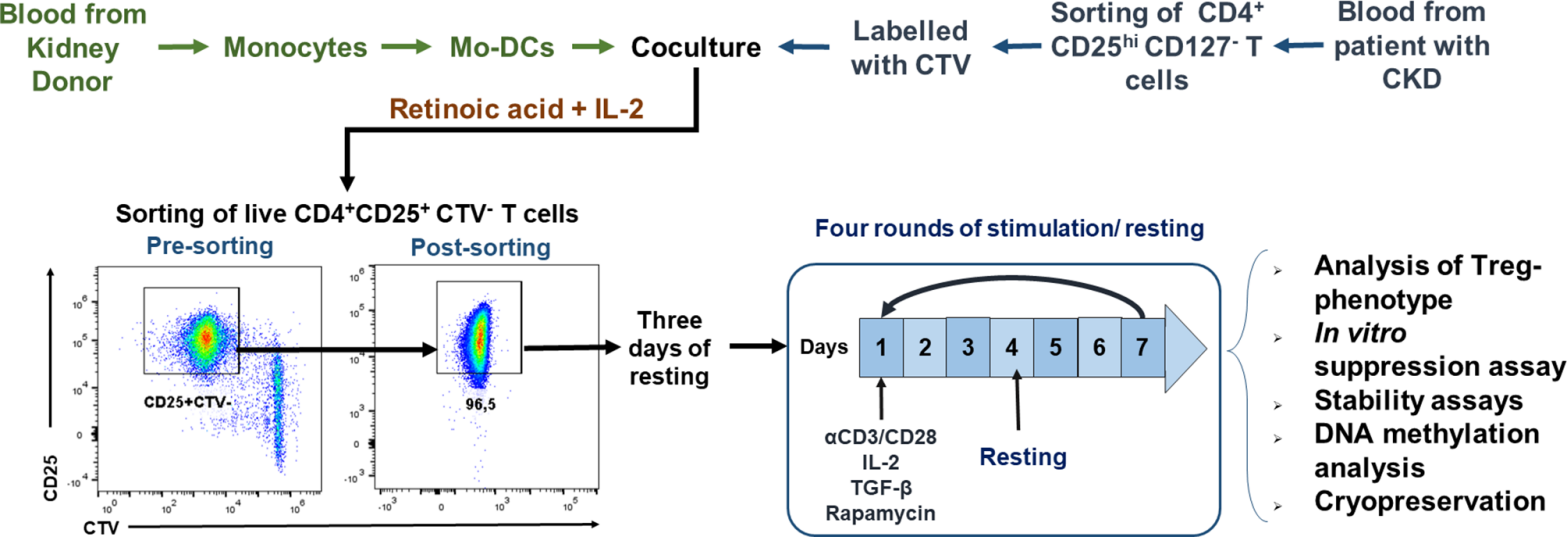
Schematic representation of the protocol used for *ex vivo* expansion of alloantigen-specific regulatory T cells (Tregs). CD4^+^CD25^hi^CD127^−^ T cells [labeled with CellTrace Violet (CTV)] sorted using fluorescence-activated cell sorting (FACS) were stimulated with allogeneic monocyte-derived dendritic cells (Mo-DCs) in the presence of IL-2 and retinoic acid. On day 7, CD4^+^CD25^hi^CTV^−^ T cells (allospecific Tregs) were purified by FACS, rested for 3 days, and then polyclonally expanded for 4 days stimulated with anti-CD3/anti-CD28, IL-2, TGF-β, and rapamycin, followed by 3 days resting with only IL-2. After four rounds of polyclonal expansion, analysis of the phenotype and *in vitro* functional assays were performed.

For the isolation of Tregs, PBMCs were stained with anti-CD4, anti-CD127, and anti-CD25 monoclonal antibodies for 20 min at 4°C in the dark, washed twice with phosphate-buffered saline (PBS), and resuspended in PBS. CD4^+^CD25^hi^CD127^−^ and CD4^+^CD25^−^CD45RA^+^ gates (**Figure S1A**) were used for sorting Tregs and naive T cells, respectively, using a BD FACSAria I cell sorter. Isolated CD4^+^CD25^hi^CD127^−^ Tregs were labeled with CTV (5 µM) according to the manufacturer’s instructions. Then, CTV-labeled Tregs (2.5 × 10^4^ cells/well) were co-cultured with irradiated allogeneic Mo-DCs (DC/Treg ratio of 1:2) for 7 days in expansion medium with 10% human AB serum, IL-2 (500 U/ml), and retinoic acid (10 nM). On day 7 of co-culture, the cells were stained with anti-CD25, anti-CD4, and Zombie Aqua™ for 20 min at room temperature in the dark. Live proliferating CD4^+^CD25^+^CTV^−^ Tregs (allospecific Tregs) (**Figure S1B**) were sorted using a MoFlo XDP cell sorter, collected in RPMI medium with 20% FBS, and cultured for 3 days in expansion medium supplemented with IL-2 (50 U/ml) plus 10% human AB serum. Then, the allospecific Tregs were polyclonally expanded using a modified protocol described previously (21, 22). Briefly, the allospecific Tregs (2.5 × 10^4^ cells/well) were stimulated with anti-CD3/anti-CD28 beads (bead/Treg ratio of 1:2) for 4 days in expansion medium with 10% human AB serum, IL-2 (300 U/ml), TGF-β (2.5 ng/ml), and rapamycin (100 nM). Then, the beads were removed with DynaMag and the cells rested for 3 days in expansion medium with IL-2 (50 U/ml) plus 10% human AB serum. Three additional rounds of stimulation/resting (7 days each) were performed (**Figure 1**). A proportion of allospecific Tregs expanded for 4 weeks were cryopreserved Section *Isolation and Cryopreservation of PBMCs*. For stability assays, on day 28 of polyclonal expansion, the Tregs were stimulated for two additional rounds of stimulation/resting with anti-CD3/anti-CD28 beads (bead/Treg ratio of 1:2) and IL-2 (100 U/ml) in the presence or absence of 10 ng/ml of IFN-γ, IL-4, IL-6, or TNF-α.

In parallel experiments, naive CD4^+^CD25^−^CD45RA^+^ T cells were polyclonally expanded using rounds of stimulation/resting with anti-CD3/anti-CD28 beads (bead/T cell ratio of 1:2) and IL-2 (300 U/ml).

### Surface and Intracellular Staining of T Cells

Expanded T cells were stained with anti-CD4, anti-CD25, anti-LAG3, anti-CD39, and Zombie Aqua™ for 20 min at room temperature in the dark and washed once with FACS buffer. For intracellular staining, Foxp3/Transcription Factor Staining Buffer Kit was used following the manufacturer’s instructions. Briefly, the cells were permeabilized with a fixation/permeabilization solution at room temperature for 1 h, washed with permeabilization buffer 1×, and incubated with anti-FOXP3, anti-CTLA-4, and anti-Helios for 30 min at 4°C in the dark. Samples were acquired on the Attune NxT Flow Cytometer and the data analyzed with FlowJo vX.0.7 software. The median fluorescence intensity (MFI) represented in the graphs was calculated by subtracting the FMO (fluorescence minus one) MFI absolute value of each sample from the respective MFI absolute value. The strategy for the analysis of Tregs by flow cytometry is presented in **Figure S1C**.

### *In Vitro* Suppression Assays

Conventional CD3^+^ T cells (Tconv) were isolated from PBMCs using the Pan T Cell Isolation Kit (Miltenyi Biotec) according to the manufacturer’s instructions, obtaining purity of CD3^+^ T cells of >85% (**Figure S2A**). For allospecific suppression assays, expanded Tregs (labeled with CTV) were co-cultured with autologous conventional CD3^+^ T cells (labeled with 5 µM CFSE, 4 × 10^4^ cells/well) at a Tconv/Treg ratio of 4:1 and the cells stimulated with irradiated allogeneic Mo-DCs (DC/T cell ratio of 1:4) in expansion medium with 10% human AB serum. For stability assays, co-cultures were stimulated in the presence or absence of 10 ng/ml of IFN-γ, IL-4, IL-6, or TNF-α. For polyclonal suppression assays, the co-cultures (at several Treg/Tconv ratios 0:1, 1:2, 1:4, 1:8, and 1:16) were stimulated with anti-CD3/anti-CD28-coated beads (bead/T cell ratio of 1:10) in RPMI medium with 10% human AB serum. After 4 days of co-culture, the cells were stained with anti-CD3, anti-CD4, and anti-CD8 for 20 min at 4°C in the dark, washed twice, and acquired on the Attune Cytometer. The data were analyzed using FlowJo vX.0.7 software. The division index (DI) was determined with CFSE dilution on gated CD4^+^ or CD8^+^ T cells, and CTV-labeled Tregs were excluded from the analysis. The strategy for the analysis of the suppression assays is present in **Figure S2B**. The percentage of suppression was calculated using the following formula:


$$\%\text{ Suppression}=\frac{\text{DI without Treg}-\text{DI with Treg}}{\text{DI without Treg}}\times 100$$

### DNA Methylation Analysis of the Treg-Specific Demethylated Region

DNA extraction and sodium bisulfite treatment were performed using the EZ DNA Methylation Direct Kit (Zymo Research Corp., Irvine, CA, USA) according to the manufacturer’s protocol. The following primers were used for the PCR amplification of bisulfite-converted genomic DNA: p-5′-TGATTTGTTTGGGGGTAGAGGATTTAGAG-3′ and o-5′-TATCACCCCACCTAAACCAAACCTACTACA-3′. PCRs were performed on thermocyclers (Thermo Fisher Scientific) in a final volume of 25 μl containing 2.5 μl PCR buffer 10×, 1 U HotStarTaq DNA Polymerase (Qiagen, Hilden, Germany), 200 μM dNTPs, 0.4 μM each of forward and reverse primers, and bisulfite-treated genomic DNA. The amplification conditions were 95°C for 15 min and 35 cycles of 95°C for 1 min, 62.5°C for 1 min, and 72°C for 1 min, and a final extension step of 10 min at 72°C. The PCR products were purified using QIAEX II gel extraction kit (Qiagen) and were cloned into a pGEM-T Easy Vector (Promega, Madison, WI, USA). DH5α competent cells were transformed with recombinant vectors and individual positive bacterial colonies were selected from which recombinant plasmid DNA was purified using FavorPrep Plasmid Extraction Mini Kit (Favorgen, Pingtung, Taiwan). The plasmid DNA was sequenced with 3500 Genetic Analyzer (Thermo Fisher Scientific) and the sequences analyzed using MEGA software v.10.0.5 (Penn State University, State College, PA, USA).

### Cytokine Production Assay

For cytokine production analysis, expanded CD4^+^ T cells (1 × 10^4^ cells/well) were stimulated with anti-CD3/anti-CD28 beads (bead/T cell ratio of 1:1) for 18 h. The levels of cytokines in the culture supernatants were measured using the kit LEGENDplex™ Human Inflammation Panel 1 13-plex (Biolegend) according to the manufacturer’s guidelines. The samples were acquired on the flow cytometer CytoFLEX (Beckman Coulter) and the data analyzed with FlowJo vX.0.7 software. Cytokine concentrations were determined using the standard curve generated in the same assay.

### Statistics

Statistical analysis was performed using GraphPad Prism v7.00 software (San Diego, CA, USA). The Shapiro–Wilk test was used to evaluate the distribution of the data. Paired and unpaired *t*-tests were used for comparing normally distributed data; Wilcoxon’s rank-sum test or the Mann–Whitney test was used for non-normally distributed data. Differences between more than two groups were calculated using one-way ANOVA or the Kruskal–Wallis test for normally or non-normally distributed data, respectively. Graphs are expressed as mean ± standard error of the mean (SEM). Values with *p* < 0.05 were considered statistically significant.

## Results

### Allospecific Tregs from CKD Patients Can Be Long-Term Expanded Showing a Highly Immunosuppressive Phenotype

CKD is a pathology characterized by progressive loss of renal function, which will eventually require renal replacement therapy, including kidney transplantation as the best alternative (1). Therefore, patients with CKD could be considered as candidates for Treg-based immunotherapy for the induction of transplant tolerance, alternatively or complementary to the use of immunosuppressive drugs. In the present study, we first evaluated the phenotype of Tregs in peripheral blood from CKD patients and healthy controls, showing similar frequencies of CD4^+^CD25^hi^CD127^−^ (**Figure S3B**) and CD4^+^CD25^hi^CD127^−^FOXP3^+^ T cells (**Figure S3C**) and no difference in FOXP3 expression (MFI values) within the CD4^+^CD25^hi^CD127^−^ T-cell population (**Figure S3D**).

With the aim of increasing the yield, purity, and viability of the cellular products obtained with current Treg-based methodologies, we designed a new experimental protocol that allows efficient long-term expansion of highly purified ^allo^Tregs from both healthy controls and CKD patients (**Figure 1**). Analysis of typical dendritic cell (DC) markers showed that the generated Mo-DCs have high expressions of CD11c, CD86, and HLA-DR and that they do not express CD14 (**Figures S4A–D**). In addition, Mo-DCs induced the *in vitro* proliferation of alloreactive CD4^+^ and CD8^+^ T cells from healthy controls at differences ratios of allo-DCs/T cells (**Figure S4E**). Then, purified CD4^+^CD25^hi^CD127^−^ Tregs were FACS sorted to a purity >95%, labeled with CTV, and co-cultured for a week with allogeneic Mo-DCs. For the initial expansion of allospecific Tregs, we used the combination of retinoic acid (RA) plus IL-2 in the co-cultures, which led to 44.8 ± 9.1% of viable proliferating cells, being CD4^+^CD25^+^CTV^−^ Tregs (^allo^Tregs) (**Figure S5A**). Parallel cultures using IL-2 plus RA alone did not induce significant proliferation of freshly purified CD4^+^CD25^hi^CD127^−^ Tregs (**Figure S5B**).

**Figure 2:**
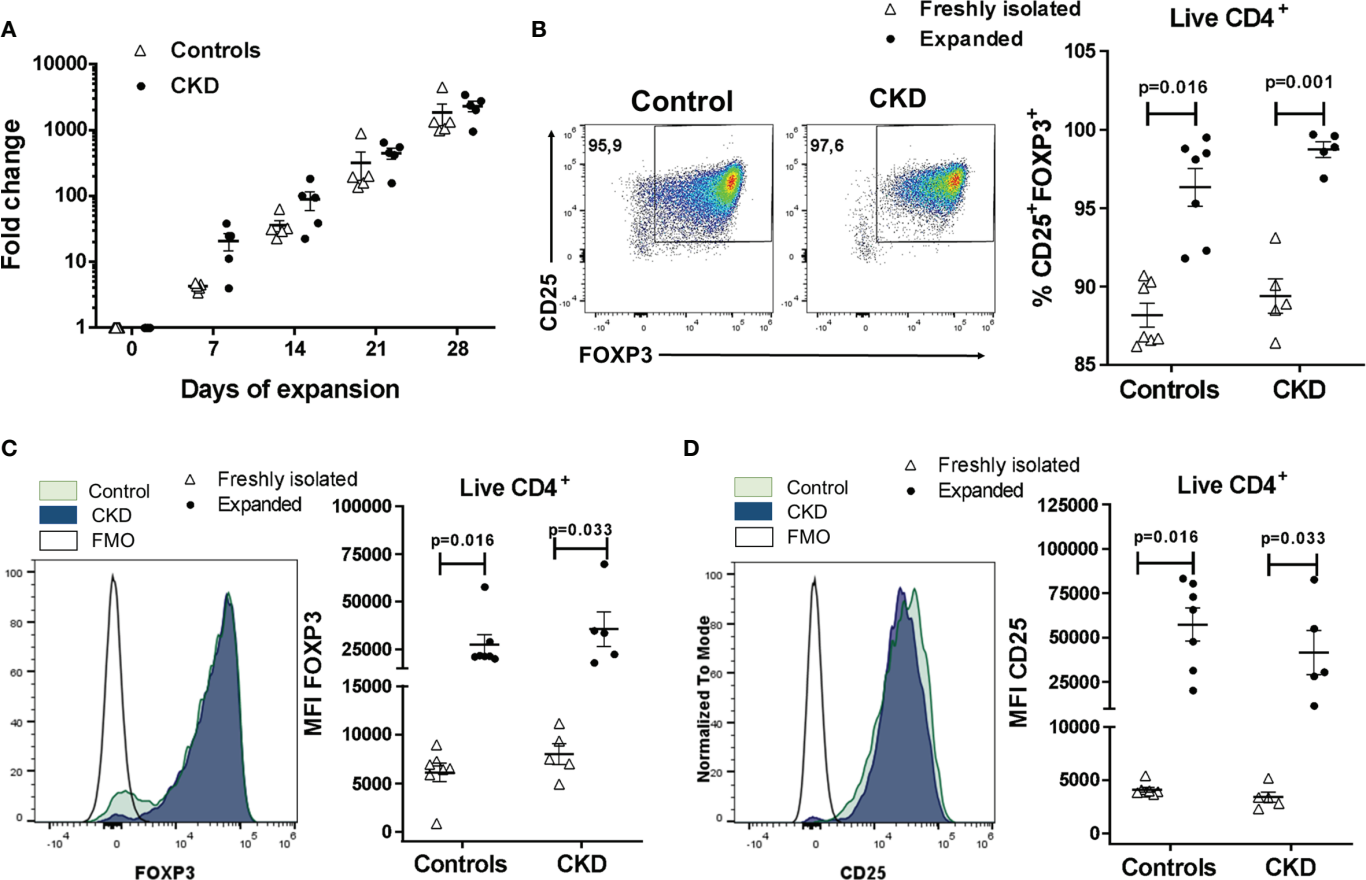
Highly purified allospecific regulatory T cells (Tregs) increased the expressions of FOXP3 and CD25 after *ex vivo* expansion. CD4^+^CD25^hi^CD127^−^ T cells [labeled with CellTrace Violet (CTV)] from healthy individuals (*white triangles*, *n* = 7) or patients with chronic kidney disease (CKD) (*black circles*, *n* = 5) were stimulated with allogeneic monocyte-derived dendritic cells (Mo-DCs). On day 7, CD4^+^CD25^hi^CTV^−^ T cells (allospecific Tregs) were purified and polyclonally expanded for 4 weeks; the expressions of FOXP3 and CD25 were evaluated by flow cytometry. **(A)** Allospecific Tregs from patients proliferated to the same extent as the Tregs from healthy controls. Fold expansion was calculated by dividing the number of Tregs obtained on the evaluated day by the number of Tregs on day 0. **(B–D)** The proportion of CD25^+^FOXP3^+^ cells **(B)** and the expressions of FOXP3 **(C)** and CD25 **(D)** were increased in the expanded alloantigen-specific Tregs (^allo^Tregs) in both study groups compared to those of freshly isolated Tregs. The median fluorescence intensity (MFI) was calculated as described in *Section 2.6*. Representative experiments are shown in **(B**–**D)**, and the *white histograms* represent FMO (fluorescence minus one) controls **(C**, **D)**. The results are shown as the mean ± SEM. Statistical analysis was performed using the Mann–Whitney *U* test or Wilcoxon’s rank-sum test.

Next, FACS-sorted allospecific Tregs from CKD patients and controls were polyclonally expanded for 4 weeks, reaching an increase in the total cell numbers from 1,800- to 2,300-fold (**Figure 2A**). Interestingly, the long-term expanded ^allo^Tregs from both groups presented a greater proliferation when they were restimulated with the DCs initially used for their expansion (donor DCs) compared to third-party DCs (**Figure S5C**), demonstrating the allospecificity of the expanded ^allo^Tregs.

Analysis of the Treg phenotype showed that the proportion of CD25^+^FOXP3^+^ cells was significantly increased in expanded ^allo^Tregs compared to freshly isolated CD4^+^CD25^hi^CD127^−^ T cells (**Figure 2B**), both in CKD patients (98.7 ± 1.1% *vs*. 89.4 ± 2.5%, *p* < 0.01) and healthy controls (96.3 ± 3.2% *vs*. 88.2 ± 2.0%, *p* < 0.05). Similarly, FOXP3 expression (**Figure 2C**) was significantly upregulated in expanded ^allo^Tregs from CKD patients (35,586 ± 20,391 *vs*. 7,986 ± 2,384, *p* < 0.05) and controls (27,390 ± 13,692 *vs*. 7,986 ± 2,384, *p* < 0.05). Likewise, the CD25 levels (**Figure 2D**) were significantly increased in ^allo^Tregs from patients (41,618 ± 27,737 *vs*. 3,399 ± 1,088, *p* < 0.05) and healthy individuals (57,421 ± 24,687 *vs*. 4,101 ± 595, *p* < 0.05). Moreover, no significant differences were found in the proportions of CD25^+^FOXP3^+^ cells and in FOXP3 and CD25 expressions in expanded ^allo^Tregs from patients compared to Tregs from controls (**Figures 2B–D**).

**Figure 3:**
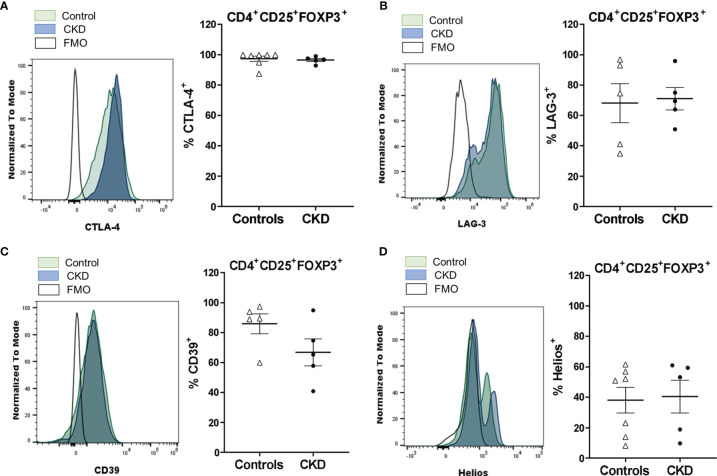
Long-term expanded alloantigen-specific regulatory T cells (^allo^Tregs) had high expressions of CTLA-4, LAG-3, and CD39. Highly purified ^allo^Tregs from healthy controls (*white triangles*, *n* = 5-7) or patients with chronic kidney disease (CKD) (*black circles*, *n* = 5) were polyclonally expanded for 4 weeks and then were stained with fluorochrome-conjugated antibodies for analysis by flow cytometry. **(A–D)** Analysis of typical Treg markers showed that the expanded ^allo^Tregs from both healthy individuals and patients with CKD had high proportions of CTLA-4^+^ **(A)**, LAG-3^+^ **(B)**, CD39^+^ **(C)**, and Helios^+^ **(D)** cells. Representative experiments are shown **(A–D)**, and the *white histograms* represent FMO (fluorescence minus one) controls. The results are shown as the mean ± SEM. Statistical analysis was performed using the Mann–Whitney *U* test. No significant differences were observed.

To further analyze the expanded Treg phenotype, we evaluated the expressions of the molecules related to the suppressive function of Tregs, such as CTLA-4, LAG-3, CD39, and Helios. CTLA-4 expression in Tregs is essential to inhibit the function of antigen-presenting cells (APCs) and the proliferation of conventional T cells *in vitro* and *in vivo* (24). Expanded ^allo^Tregs from CKD patients showed a high proportion of CTLA-4^+^ cells, similarly to ^allo^Tregs from controls (**Figure 3A**). Furthermore, no significant differences were found in the expression levels of CTLA-4 in the long-term expanded ^allo^Tregs from patients compared to the ^allo^Tregs from healthy individuals (**Figure S6A**). LAG-3 is an immunoglobulin superfamily member that has a high affinity to MHC class II molecules expressed on APCs, and this interaction inhibits both the maturation and the antigen-presenting capacity of DCs (25). The expression levels of LAG-3 observed in expanded ^allo^Tregs from patients were as high as those of ^allo^Tregs from healthy controls (**Figure 3B** and **Figure S6B**). CD39 is an ectonucleotidase involved in the generation of pericellular adenosine, which inhibits the function of conventional T cells and DCs through the activation of the adenosine 2A receptor (26). Our data showed a high proportion of CD39^+^ cells in the long-term expanded ^allo^Tregs from CKD patients and healthy controls (**Figure 3C**). Moreover, no significant differences were found in the CD39 expression of the expanded ^allo^Tregs from patients compared to the Tregs from controls (**Figure S6C**). Helios is a member of the Ikaros transcription factor family that is important for Treg function (27). The expanded ^allo^Tregs from both patients and controls displayed heterogeneous proportions of Helios^+^ cells (**Figure 3D**) and levels of this transcription factor (**Figure S6D**).

Interestingly, the expanded ^allo^Tregs from healthy donors showed increased levels of FOXP3 (**Figure S7A**) and higher proportions of CD25^+^FOXP3^+^ (S7B), CTLA-4^+^ (S7C), Helios^+^ (S7D), and LAG-3^+^ (S7E) cells compared to expanded naive CD4^+^ T cells (Tn). To obtain a more detailed profile of the expanded Tregs, we also evaluated cytokine production; importantly, ^allo^Tregs do not produce significant levels of inflammatory cytokines compared to expanded Tn (**Figure S8**).

### Expanded ^allo^Tregs Efficiently Suppress the Proliferation of Conventional T Cells in an Alloantigen-Specific Manner

**Figure 4:**
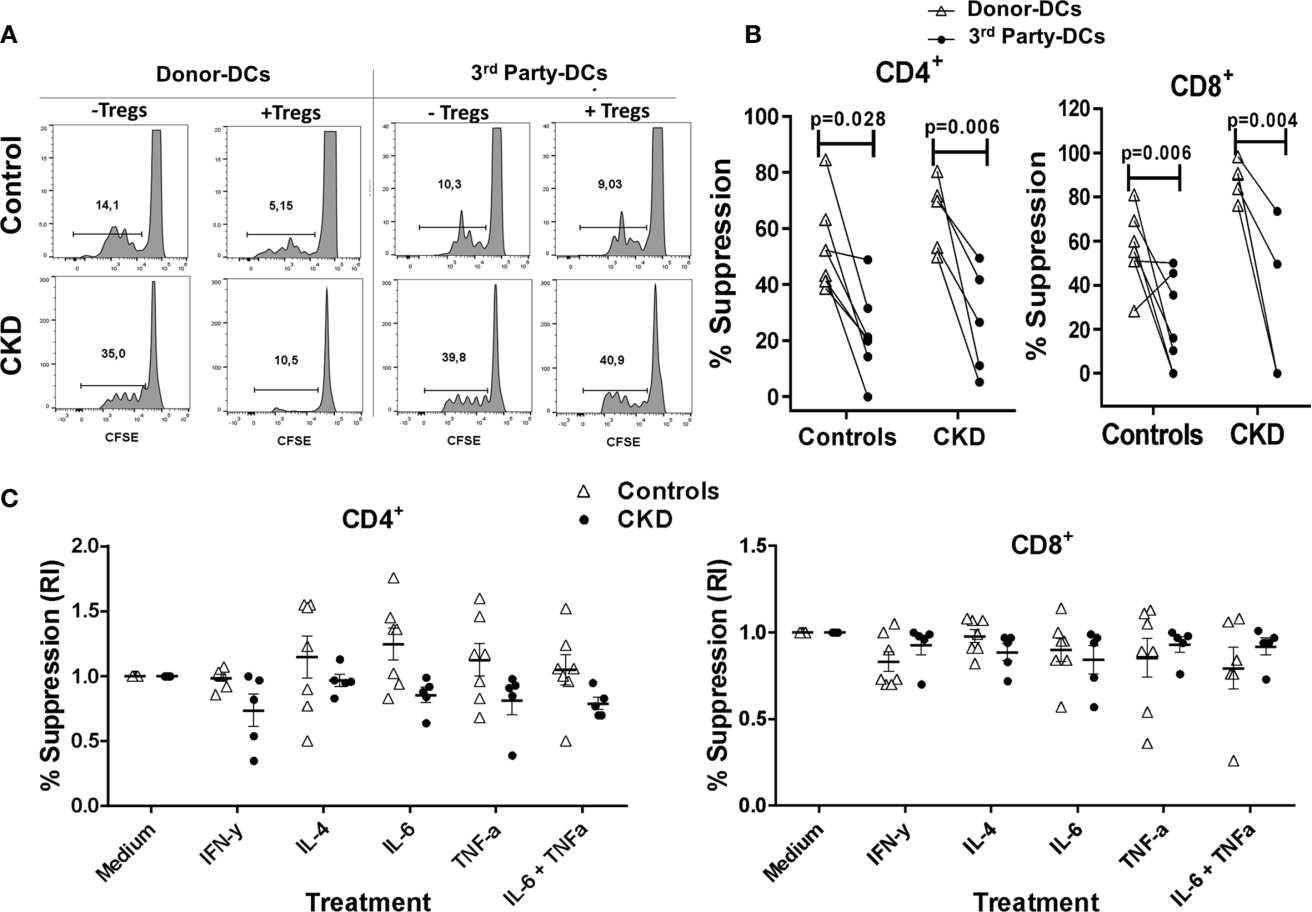
Expanded allospecific regulatory T cells (Tregs) suppressed the proliferation of conventional T cells in an alloantigen-specific manner. Expanded allospecific Tregs (^allo^Tregs) from healthy individuals (*white triangles*, *n* = 7) or patients with chronic kidney disease (CKD) (*black circles*, *n* = 5) were co-cultured with conventional CD3^+^ T cells [labeled with carboxy fluorescein succinimidyl ester (CFSE)] and stimulated with allogeneic monocyte-derived dendritic cells (Mo-DCs) from their respective donors or from non-related individuals (third party). On day 4 of culture, T-cell proliferation was evaluated by flow cytometry. **(A)** Representative experiment. **(B)** Expanded ^allo^Tregs suppressed the proliferation of both CD8^+^ and CD4^+^ conventional T cells (Tconv) only when they were stimulated with the dendritic cells (DCs) with which they were initially expanded (DCs from donor), but not when they were stimulated with unrelated DCs (third party). **(C)** ^allo^Tregs efficiently suppressed alloreactive T cells in the presence of the inflammatory cytokines IFN-γ, IL-4, IL-6, and TNF-α **(C)**. The relative increase (IR) of the percentage of suppression was calculated by dividing the value in the presence of the cytokine by the value in the absence of cytokine (medium). All experiments were performed in duplicate. The results are shown as the mean ± SEM. Statistical analysis was performed using unpaired *t*-test **(B)** or one-way ANOVA **(B)**.

After successful expansion of ^allo^Tregs, we evaluated their suppressive capacity *in vitro* (see *Materials and Methods*). As shown in **Figure 4A**, the expanded ^allo^Tregs suppressed the proliferation of alloreactive CD3^+^ Tconv, only when they were stimulated with the DCs toward which they were initially expanded (donor), but not in the presence of DCs from a third party. Moreover, the ^allo^Tregs from both groups significantly inhibited the proliferation of both CD4^+^ and CD8^+^ T cells (**Figure 4B**). Of note is that the allospecific Tregs from CKD patients suppressed the proliferation of alloreactive Tconv to the same extent as that of the Tregs from controls (**Figure 4B**).

Under inflammatory conditions, such as autoimmune diseases and allergy, it has been shown that several cytokines (IL-4, TNF-α, and IL-6) may be involved in downmodulation of Treg suppression (28–30). Thus, it is crucial to assess the functional stability of Tregs for therapeutic purposes. Therefore, we next evaluated the suppressive function of the expanded ^allo^Tregs *in vitro* in the presence of inflammatory cytokines. As shown in **Figure 4C**, the expanded ^allo^Tregs from CKD patients and healthy individuals maintained their ability to inhibit the alloantigen-specific proliferation of CD4^+^ (left) and CD8^+^ (right) T cells under all inflammatory conditions used. Analysis of the proliferation of Tconv without Tregs in the presence of cytokines showed no significant differences compared to Tconv with media alone (data not shown).

### Long-Term Expansion Results in Reduced Demethylation of TSDR-Foxp3 in ^allo^Tregs

**Figure 5:**
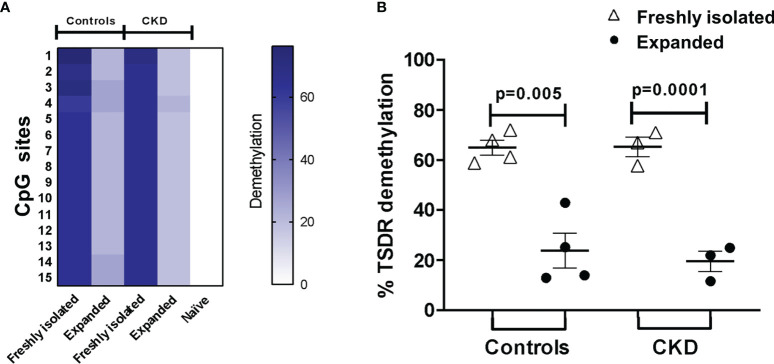
Long-term expansion reduced the demethylation of TSDR-*Foxp3* in purified allospecific regulatory T cells (Tregs). CpG methylation analysis of the Treg-specific demethylated region (TSDR) in the *Foxp3* gene was evaluated in purified CD4^+^CD25^hi^CD127^−^ Tregs (freshly isolated) and in expanded alloantigen-specific Tregs (^allo^Tregs). **(A)** Each *square* represents one CpG site analyzed (of a total of 15 CpGs). Six to seven clones per DNA sample were sequenced. The demethylation color code ranges from *white* (0% demethylation) to *navy blue* (80% demethylation), according to the color scale. Purified CD4^+^CD25^−^CD45RA^+^ T cells (naive) displayed a completely methylated TSDR and were used as the negative control. **(B)** Freshly isolated Tregs from both groups displayed high demethylation of TSDR-*Foxp3*, while expanded ^allo^Tregs showed a significant increase in the methylation of the TSDR. DNA samples from three male chronic kidney disease (CKD) patients and four male healthy controls were analyzed. The results are expressed as the mean ± SEM. Statistical analysis was performed using paired *t*-test.

The stability of FOXP3 expression has been shown to correlate with the increased demethylation of the Treg-specific demethylated region (TSDR) of the *Foxp3* locus (31). In an attempt to investigate whether the observed phenotype in Tregs was associated with their epigenetic status, we evaluated the methylation of CpG sites in the *Foxp3* gene. Unexpectedly, even though the expanded ^allo^Tregs showed high FOXP3 expression and suppressive function, the percentage of demethylation of TSDR-*Foxp3* was lower in the expanded Tregs from both groups compared with that in freshly isolated Tregs (**Figure 5**).

### ^allo^Tregs Maintain a Suppressive Phenotype After Expansion Under an Inflammatory Microenvironment

**Figure 6:**
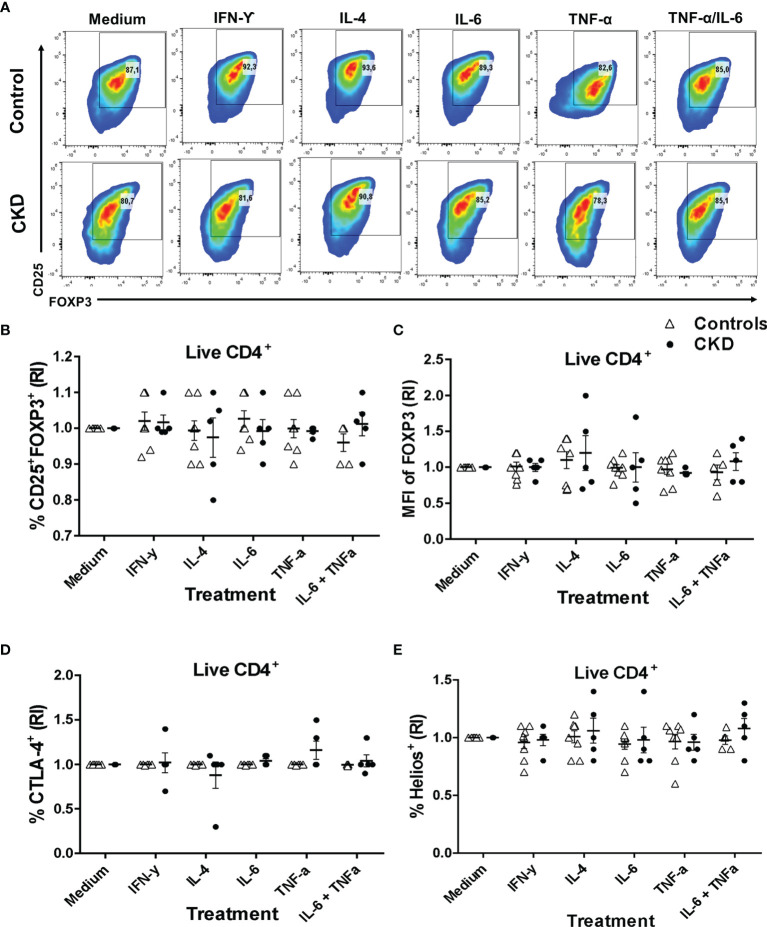
Expanded alloantigen-specific regulatory T cells (Tregs) maintain their immunosuppressive phenotype after stimulation in an inflammatory microenvironment. Long-term expanded Tregs from healthy controls (*white triangles*, *n* = 7) or patients with chronic kidney disease (CKD) (*black circles*, *n* = 5) were stimulated with anti-CD3/anti-CD28 in the presence or absence of inflammatory cytokines (IFN-γ, IL-6, IL-4, or TNF-α) for two additional weeks. The expressions of Treg markers were determined by flow cytometry. **(A–D)** Activation of allospecific Tregs from CKD patients in the presence inflammatory cytokines did not have an effect on the percentages of CD25^+^FOXP3^+^ **(A, B)**, CTLA-4^+^ **(D)**, and Helios^+^ **(E)** cells and on the expression of FOXP3 **(C)** to the same extent as the Tregs from healthy controls. A representative experiment is shown in **(A)**. The relative increase (IR) of the percentage or median fluorescence intensity (MFI) was calculated by dividing the value in the presence of the cytokine by the value in the absence of cytokines (medium). The results are shown as the mean ± SEM. Statistical analysis was performed using the Kruskal–Wallis test. No significant differences were observed.

One major concern regarding the use of Tregs for immunotherapy is the risk of their conversion into inflammatory T cells and the loss of the suppressive activity during inflammatory responses (32). To gain insight into the stability of Tregs, we evaluated both the phenotype and the *in vitro* function after two additional weeks of polyclonal stimulation with only IL-2 in the presence or absence of inflammatory cytokines. Importantly, the proportion of CD25^+^FOXP3^+^ cells (**Figures 6A, B**) and the expressions of FOXP3 (**Figure 6C**) and CD25 (**Figure S9A**) in the expanded ^allo^Tregs from both patients with CKD and healthy controls were maintained in the presence of all inflammatory cytokines evaluated (IFN-γ, IL-4, IL-6, and TNF-α). Moreover, the stimulation in the presence of inflammatory cytokines did not affect the proportions (**Figures 6D, E**) or the expression levels (**Figures S9B, C**) of CTLA-4^+^ (**Figure 6D**) and Helios^+^ (**Figure 6E**) in the expanded ^allo^Tregs from both groups. Notably, the expanded ^allo^Tregs from controls and CKD patients maintained >80% FOXP3^+^CD25^+^ (**Figure S9D**) and did not significantly reduce their FOXP3 expression levels in the absence of TGF-β and rapamycin (**Figure S9E**).

**Figure 7:**
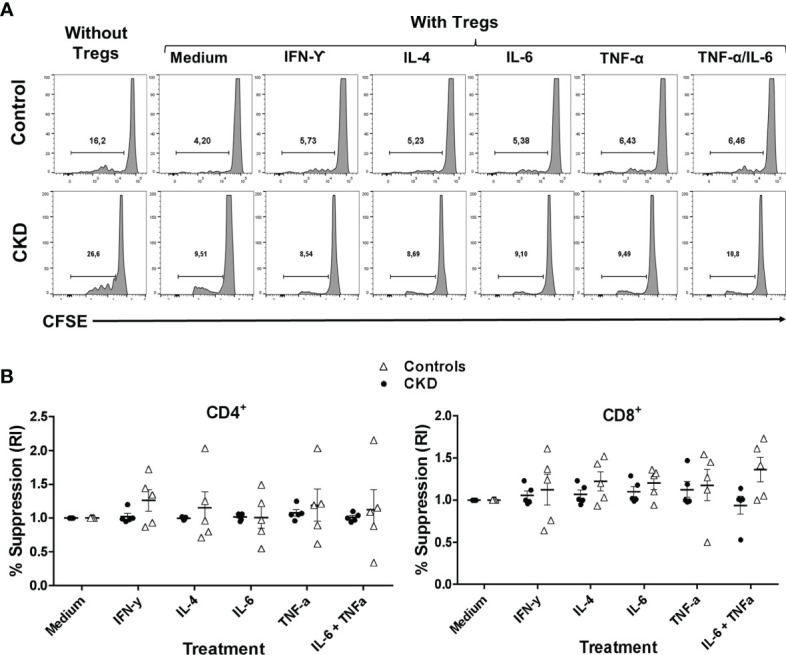
Allospecific regulatory T cells (^allo^Tregs) maintained their *in vitro* function after stimulation in an inflammatory microenvironment. Long-term expanded ^allo^Tregs from healthy individuals (*white triangles*, *n* = 5) or patients with chronic kidney disease (CKD) (*black circles*, *n* = 5) were stimulated with anti-CD3/anti-CD28 in the presence or absence of inflammatory cytokines for two additional weeks, and then *in vitro* allospecific suppression assays were performed. **(A, B)** The ^allo^Tregs from both patients and healthy controls maintained their capacity to suppress both CD4^+^ (*left*) and CD8^+^ (*right*) T-cell proliferation after expansion in the presence of all the inflammatory cytokines evaluated (IFN-γ, IL-6, IL-4, and TNF-α). A representative experiment is shown in **(A)**. The relative increase (IR) of the percentage of suppression was calculated by dividing the value in the presence of the cytokine by the value in the absence of cytokines (medium). All experiments were performed in duplicate. The results are shown as the mean ± SEM. Statistical analysis was performed using the Kruskal–Wallis test. No significant differences were observed.

Subsequently, we evaluated whether the suppressive phenotype of the stimulated Tregs in an inflammatory microenvironment correlated with their *in vitro* function. Indeed, the allospecific Tregs from CKD patients and controls similarly inhibited the proliferation of both CD4^+^ and CD8^+^ alloreactive T cells after they were polyclonally expanded for 2 weeks in the presence of inflammatory cytokines (**Figure 7**).

### Allospecific Tregs Maintain Their Immunosuppressive Phenotype After Long-Term Cryopreservation

**Figure 8:**
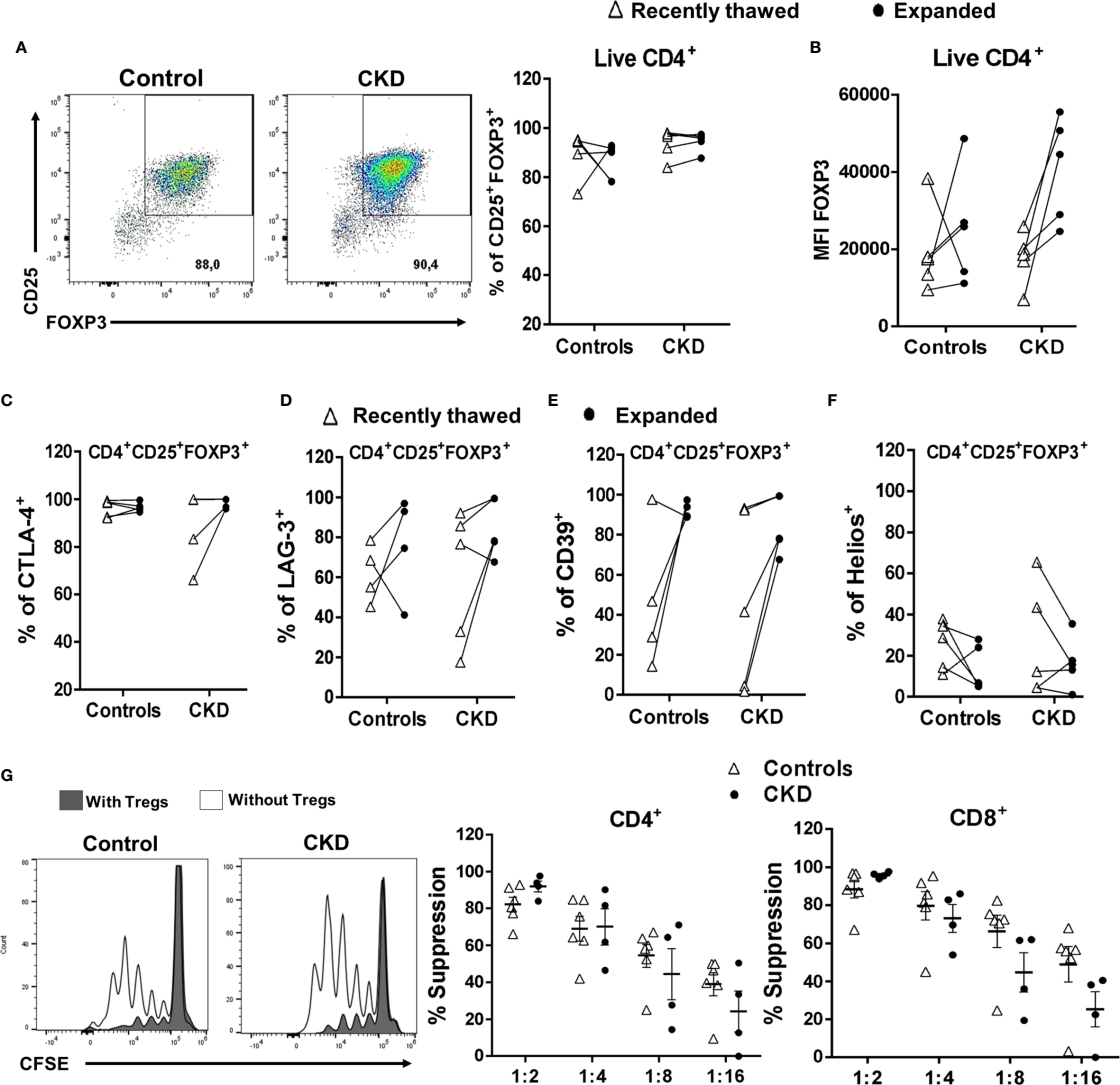
Expanded regulatory T cells (Tregs) maintained their phenotype and *in vitro* function after long-term cryopreservation. On day 28 of polyclonal expansion, alloantigen-specific Tregs (^allo^Tregs) from healthy individuals (*white triangles*, *n* = 4) or patients with chronic kidney disease (CKD) (*black circles*, *n* = 4) were cryopreserved for a long time (>6 months), and then the cells were thawed and polyclonally expanded. The expressions of the Tregs markers and their *in vitro* immunosuppressive function were determined by flow cytometry. **(A–F)** After 2 weeks of stimulation, thawed ^allo^Tregs from both groups maintained the proportions of CD25^+^FOXP3^+^ **(A)**, CTLA-4^+^ **(C)**, LAG-3^+^ **(D)**, and CD39^+^ **(E)** cells and the expression of FOXP3 **(B)**, but a low proportion of Helios^+^ cells **(F)**. **(G)** Cryopreserved allospecific Tregs can suppress the proliferation of both polyclonal CD4^+^ (*left*) and CD8^+^ (*right*) T cells after 2 weeks of expansion. Representative experiments are shown in **(A, G)**. The results are shown as the mean ± SEM. Statistical analysis was performed using the Mann–Whitney *U* test or Wilcoxon test. No significant differences were observed.

Finally, we investigated the effect of cryopreservation on the phenotype and function of expanded allospecific Tregs. For this aim, at the end of the expansion, the allospecific Tregs were cryopreserved for a long time (>6 months), and then the cells were thawed and polyclonally expanded. Cryopreserved ^allo^Tregs from both patients and controls were successfully expanded for 2 weeks, showing higher percentage of CD25^+^FOXP3^+^ cells (**Figure 8A**) and levels of FOXP3 (**Figure 8B**). In addition, the ^allo^Tregs from both groups have similar percentages of CTLA-4^+^ (**Figure 8C**), LAG-3^+^ (**Figure 8D**), and CD39^+^ (**Figure 8E**) cells. Interestingly, when we compared the freshly thawed and expanded Tregs, we found that some suppressive markers (FOXP3, LAG-3, CTLA-4, and CD39) were enhanced after expansion (**Figures 8B–E**). However, the proportion of Helios^+^ cells was slightly lower in cryopreserved expanded Tregs (**Figure 8F**) compared to that in recently thawed Tregs. In this context, we and others have previously reported the decrease of Helios after *in vitro* stimulation (21, 33).

**Figure 9:**
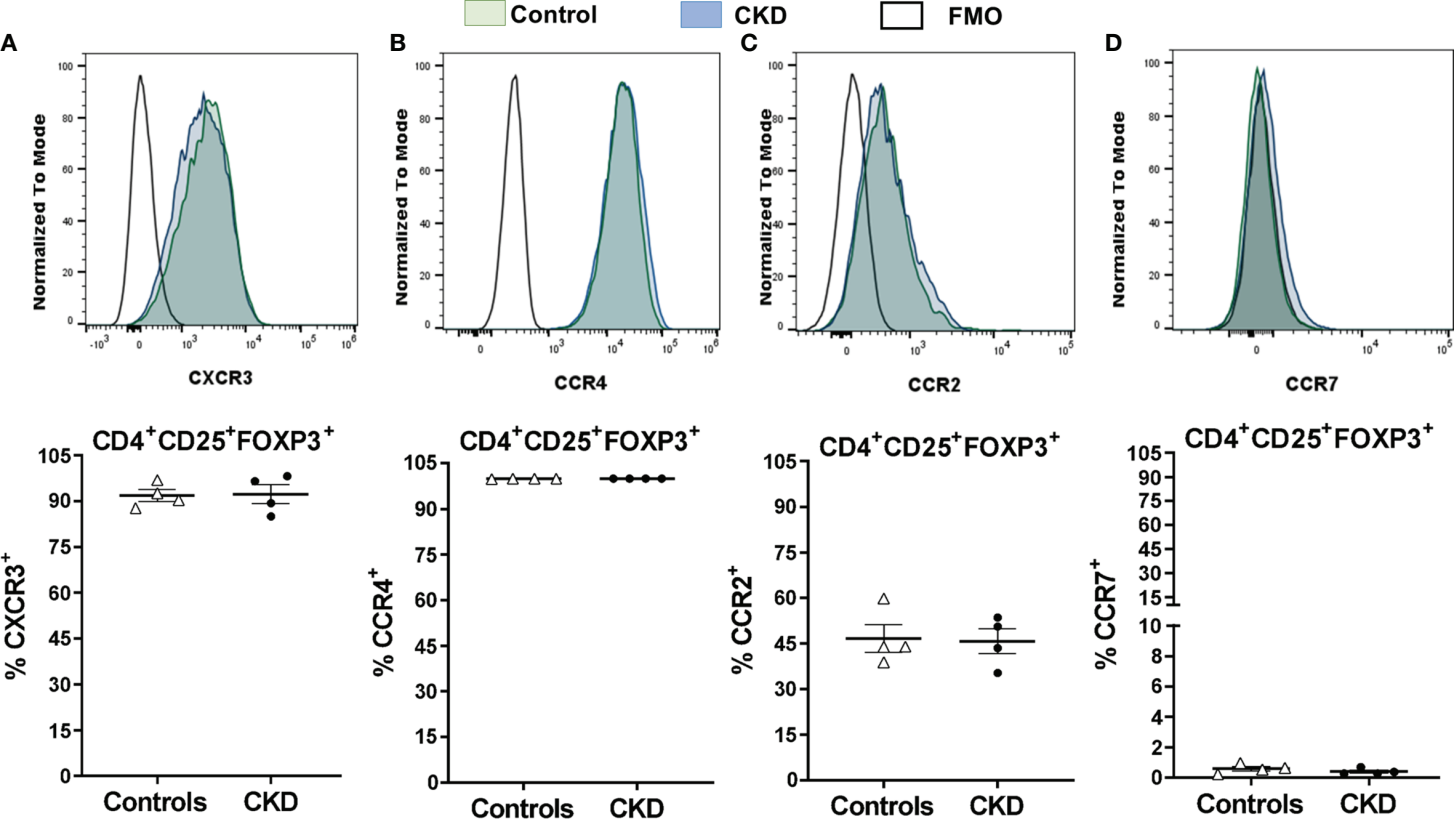
Cryopreserved allospecific regulatory T cells (^allo^Tregs) expressed high levels of the chemokine receptors CXCR3, CCR4, and CCR2. Long-term cryopreserved ^allo^Tregs from healthy controls (*white triangles*, *n* = 4) or patients with chronic kidney disease (CKD) (*black circles*, *n* = 4) were thawed and polyclonally expanded for 2 weeks. The expressions of chemokine receptors were determined by flow cytometry. **(A, B)** The expanded Tregs of both groups had high percentages of CXCR3^+^ **(A)**, CCR4^+^ **(B)**, and CCR2^+^ **(C)** cells, but a low proportion of CCR7^+^ cells **(D)**. Representative experiments are shown in **(A–D)**. *White histograms* represent FMO (fluorescence minus one) controls. The results are shown as the mean ± SEM. Statistical analysis was performed using unpaired *t*-test. No significant differences were observed.

After successful expansion of cryopreserved Tregs, we investigated the *in vitro* function of these cells. Allospecific Tregs from patients with CKD showed high ability to suppress the proliferation of polyclonal CD4^+^ and CD8^+^ T cells, similarly to Tregs from healthy controls, at all evaluated ratios (**Figure 8G**). Moreover, analysis of the expression of chemokine receptor demonstrated that expanded ^allo^Tregs from both CKD patients and controls showed high expressions of CXCR3, CCR4, and CCR2, recently reported to play an important role in Treg graft homing (23), while ^allo^Tregs displayed very low levels of CCR7 in both groups (**Figure 9**).

## Discussion

In this work, we addressed key issues previously considered to ensure the efficacy and safety of allospecific Treg therapy, including Treg expansion, purity, and stability. To increase the yield of ^allo^Tregs, FACS-sorted CD4^+^CD25^hi^CD127^−^ Tregs were expanded in the presence of allogeneic DCs plus RA and IL-2, which increased the numbers of purified ^allo^Tregs compared to previous reports using rapamycin in primary co-cultures (**Figure S5A**) (18). A previous study had shown that the addition of RA favors the maintenance of the expression of FOXP3 in short-term expanded Tregs (34). In this context, RA significantly promoted the accumulation of H3K9Ac and H3K4me3 in the promoter region and partially decreased the methylation of CpG in the central nervous system (CNS) regions of the *Foxp3* gene locus (35). Moreover, RA prevented FOXP3 degradation by downregulating E3 ligase Stub1 expression in activated natural Tregs (35).

To ensure the high purity of ^allo^Tregs, proliferating Tregs (CD4^+^CD25^+^CTV^−^) were isolated by FACS, obtaining a purity of >95% (data not shown). Although most of the current protocols used in Treg immunotherapy are based on immunomagnetic separation, in compliance with good manufacturing practices (GMP) [reviewed in (8)], this process has the limitation of not allowing the use of multiple parameters, including cell proliferation dyes. Additionally, the FACS-based isolation of Tregs has been implemented in clinical trials (36, 37), demonstrating the safety of the cellular product obtained with this approach.

Extensive *in vitro* Treg expansion could result in the loss of FOXP3 expression (20). Previous reports have shown that rapamycin allows the preferential growth of Tregs and maintenance of FOXP3 expression (38, 39), while TGF-β has been linked to the upregulation of FOXP3 through epigenetic modification in the *Foxp3* locus (40, 41). To favor the maintenance of FOXP3 expression during the polyclonal expansion of ^allo^Tregs, we added both rapamycin and TGF-β to our *in vitro* cultures, obtaining >95% of CD25^+^FOXP3^+^ Tregs after 4 weeks of expansion. With this protocol, we achieved an expansion from 1,800- to 2,300-fold ^allo^Tregs after 4 weeks of culture (**Figure 2A**), while previous studies reported an expansion ranging from 8- to 780-fold for 12–42 days of culture [revised in (19)]. These results are similar to those obtained in the expansion of allospecific iTregs using a slightly modified protocol, favoring the enrichment of FOXP3^+^ iTregs (from 60% to >90% of FOXP3^+^ cells) (22). Moreover, expanded ^allo^Tregs displayed an increase in the expressions of CD25 and CTLA-4, in correlation with the increase of FOXP3, which directly upregulated the transcription of both molecules by binding to the *Il2ra* and *Ctla4* loci (42). This is functionally relevant as, in the context of transplantation, it has been established that CTLA-4 signaling participates in the early induction of allograft acceptance (24, 43). Importantly, >95% of our expanded ^allo^Tregs expressed high levels of CTLA-4, while in a previous report (18) only 15% of the ^allo^Tregs obtained were CTLA-4^+^, although the stimulation and staining conditions used in these two studies could account for the differences observed.

Moreover, the ^allo^Tregs contained high proportions of positive cells for LAG-3 and CD39, which are characteristic suppressive markers of this subpopulation. In the setting of allotransplant, the upregulation of LAG-3 and CD39 on Tregs contributed to prolonging the survival of allografts by modulating the production of inflammatory cytokines in Tconv and the function of DCs (44–47). On the other hand, the ^allo^Tregs from both patients and healthy controls displayed a heterogeneous expression of Helios, a transcription factor that is required in Tregs to induce the expression of Treg-related genes (48), enhance suppressive function (49), and maintain a stable phenotype during inflammatory responses (50). Although some ^allo^Tregs showed low expressions of Helios, we (21) and others (33) have described that expanded Tregs may retain their suppressive function despite a reduced Helios expression, suggesting that this marker may not necessarily define the functional status of Tregs.

As some Treg markers can be transiently expressed by activated T cells (51–53), we also analyzed parallel cultures using activated naive CD4^+^ T cells. Most importantly, ^allo^Tregs showed highly increased levels of FOXP3 (eightfold), CTLA-4 (sevenfold), and LAG-3 (threefold) compared to those in expanded Tn cells, while Helios was only significantly detected in ^allo^Tregs (**Figure S7**). Furthermore, to discard that this phenotype was not a consequence of T-cell receptor activation, we evaluated the expressions of the Treg markers after a period of resting. These data support the notion that Treg identity is maintained in our long-term expanded cultures.

To further analyze the profile of our ^allo^Tregs, we evaluated cytokine production, as previous studies have described the detection of inflammatory cytokines in expanded, non-purified allospecific Tregs (54). It was shown that our ^allo^Tregs were unable to significantly release inflammatory cytokines. This discards the possibility of contamination of the activated Tconv or pro-inflammatory Treg conversion in our expanded cultures.

The expressions of immunoregulatory markers were in agreement with the efficient alloantigen-specific suppression of Tconv (>50%) using a 1:4 ratio (Treg/Tresp). This is in agreement with previous studies showing that the addition of rapamycin in cultures enhances the suppressive function of Tregs (38, 39). On the other hand, although the percentage of suppression achieved by our ^allo^Tregs appeared to be lower than that previously reported, where the allospecific CFSE^−^ Tregs were also purified (18), in this study, the expanded Tregs did not have a resting period prior to the suppression assay, and the authors performed the suppression assays based on using ^3^H thymidine incorporation, which may have led to overestimation of the results (55). In another study, allospecific Tregs were able to effectively suppress responder T cells at a ratio of 1:100 (56); however, the authors used CD4^+^CD25^−^ sorted T lymphocytes instead of the CD3^+^ T cells used in our experiments, and purified peripheral blood DCs were used instead of Mo-DCs. These differences make the suppression indexes less comparable.

Under inflammatory conditions, several studies have shown that IL-6, IL-4, IL-12, and TNF-α drive the loss of FOXP3 expression and, therefore, the suppressive capacity of Tregs (57–59). Alternatively, the exposure of Tregs to an inflammatory microenvironment may have promoted the co-expression of T helper (Th)-specific transcription factors that are key for Treg specialization and homing to inflammatory sites, including the allograft (23). Our data showed that ^allo^Treg suppression under a pro-inflammatory milieu was similar to that in the absence of inflammatory cytokines (**Figure 4**). However, in these experiments, we cannot exclude the effect of cytokines on responder T cells, in addition to Tregs; this may explain the high heterogeneity of the responses observed among patients. Therefore, we also explored the effect of cytokines directly on Tregs after a 2-week activation period in the presence of the same stimuli. Interestingly, ^allo^Tregs maintained high levels of FOXP3 and the characteristic markers, including those of Helios and CTLA-4 (**Figure 6**), which correlated with their suppressive function (**Figure 7**). Therefore, despite the long-term expansion of ^allo^Tregs, they appeared to have maintained their phenotype and suppressive function under inflammatory conditions. Our study is in line with a previous report showing the effect of rapamycin on Treg stability through preventing the production of pro-inflammatory cytokines in expanded Tregs and inhibiting the conversion of Tregs toward an inflammatory phenotype (60). In addition, the expansion of allospecific nTregs in the presence of IL-2 alone was able to maintain >80% FOXP3^+^ (**Figure S9D**), while both the induction and the maintenance of FOXP3 in expanded allospecific iTregs (22) and polyclonal iTregs (61) were shown to be highly dependent on the presence of TGF-β and rapamycin in the *in vitro* cultures.

The stability of FOXP3 expression has been shown to correlate with the complete demethylation of the TSDR within the *Foxp3* locus (31). The long-term expanded ^allo^Tregs from both groups showed lower TSDR demethylation compared to that of freshly isolated Tregs. This is an unexpected result, as TGF-β has been linked to the epigenetic stabilization of FOXP3 expression through the inhibition of DNMT1 and Uhrf1 (40, 41). IL-2R signaling promoted the recruitment of TET2 (a methylcytosine dioxygenase that catalyzes the demethylation of cytosines) to TSDR and maintained the demethylated CpG sites in the *Foxp3* locus (62, 63). Therefore, the increase in FOXP3 expression and suppressive function of the expanded ^allo^Tregs did not correlate with the demethylation status of the *Foxp3* locus. Such discordance has been previously reported by our group in both expanded iTregs (22) and nTregs (21). Moreover, another study showed that the hypomethylation of TSDR in Tregs from juvenile idiopathic arthritis was not associated with their FOXP3 levels (64). Interestingly, it has been shown that targeted demethylation of *Foxp3-*TSDR does not ensure the stable suppressive function in FOXP3-induced primary T cells (65), indicating that TSDR demethylation by itself is not sufficient for Treg lineage commitment, although it still may be necessary for Treg stability.

The discrepancies observed between the demethylation of TSDR and the expression of FOXP3 might be explained by the fact that other potential epigenetic mechanisms or posttranslational modifications can regulate the identity of Tregs [revised in (66, 67)]. In this context, it has been shown that the loss of FOXP3 expression induced by the increase of CNS2 methylation in the *Foxp3* locus, observed under inflammatory conditions, can be counteracted by the recruitment of methyl-CpG binding protein 2 (MeCP2), an X-linked multifunctional epigenetic regulator, to *Foxp3*-CNS2, which in turn induces histone H3 acetylation, leading to stable FOXP3 expression (68). Additionally, the use of TGF-β in our cultures may have promoted the phosphorylation (69) and acetylation (70) of FOXP3 on multiple amino acid residues, thus reducing its ubiquitination and proteasomal degradation.

Additionally, as TSDR is still being used as the main marker for Treg stability for immunotherapy in the clinic, several approaches have been proposed to ensure the long-term functionality of Tregs *in vivo* for their effective use in immunotherapy. In this context, as an attempt to preserve *Foxp3* demethylation in the Treg cellular product used for immunotherapy, a recent report has demonstrated that a CD70^−^CD27^+^ population isolated from *in vitro* expanded polyclonal CD4^+^CD25^+^ Tregs gave rise to Tregs with a highly hypomethylated TSDR (71). In addition, the purification of long-term activated Tregs, based on CD137^+^CD154^−^ markers, allowed the *in vitro* expansion of Tregs with an epigenetic signature that is associated with functional stability (72).

Cryopreservation of Tregs offers several advantages for personalized immunotherapy, including their long-term storage and flexible timing and dosage of Treg infusion (8). However, whether this process affects the viability and/or stability of Tregs remains controversial. In this context, some reports have shown a reduction in the frequency of FOXP3^+^ cells in cryopreserved PBMCs (73, 74), while other reports showed no significant differences between cryopreserved and freshly isolated Tregs (75). Similarly, previous studies (76, 77) have shown that the expressions of the markers CD25 and FOXP3 in Tregs, as well as their suppressive function, were readily affected after thawing. Interestingly, the restimulation of these Tregs was able to restore their phenotype and function (76, 77). In the present work, we found that the cryopreservation of *in vitro* expanded ^allo^Tregs preserved their FOXP3 expression immediately after thawing and, more importantly, that ^allo^Tregs can be further expanded, reaching the numbers required for Treg adoptive cell therapy.

The chemokine receptor (CCR) expression profiles in Tregs may enhance the suppression of the alloreactive populations in order to establish efficient allograft tolerance (23). Our results showed that the cryopreserved ^allo^Tregs expressed high levels of CCR4 and CXCR3 after two rounds of expansion, in agreement with a previous study (78) showing that both chemokine receptors were upregulated in Tregs stimulated in the presence of rapamycin. CXCR3^+^ Tregs can efficiently restrict Th1 immune responses (79), while specialized memory CCR4^+^ Tregs inhibited the Tconv proliferation by a FasL-dependent mechanism (80). Importantly, the role of infiltrating CXCR3^+^IL-10^+^TGF-β^+^ Tregs was demonstrated in a kidney transplant mouse model, where the deletion of these cells led to allograft rejection (5). Finally, CCR2 expression in our expanded ^allo^Tregs may be biologically relevant, as this receptor has been involved in Treg homing to both draining lymph nodes and allograft, thereby promoting the suppression of inflammatory T-cell responses, as demonstrated in an islet transplantation model (81).

In conclusion, we demonstrate that ^allo^Tregs can be efficiently purified and expanded, maintaining a suppressive phenotype, most importantly from patients with CKD, who are candidates for kidney transplantation. The functionality shown after cryopreservation demonstrated the feasibility of the long-term storage of this cellular product and supports their potential use for personalized Treg therapy in transplanted patients. However, it is important to further investigate the epigenetic and posttranslational mechanisms underlying the FOXP3 expression and suppressive function maintained by our expanded ^allo^Tregs and to explore whether recent protocols successfully used in the selection and expansion of polyclonal Tregs (71, 72) can be applied to ^allo^Tregs in order to prove whether an epigenetic signature and/or phenotype can help identify the most appropriate Tregs for immunotherapy.

## Data Availability Statement

The raw data supporting the conclusions of this article will be made available by the authors, without undue reservation.

## Ethics Statement

The studies involving human participants were reviewed and approved by the Research Ethics Committee, Instituto Nacional de Ciencias Medicas y la Nutricion Salvador Zubiran (#1831). The patients/participants provided written informed consent to participate in this study.

## Author Contributions

AC-H performed the experiments, analyzed the data, and wrote the draft of the manuscript. EA-S, SA-C, KR-C, and NL performed the experiments. EA-S and SA-C wrote sections of the manuscript. JA contributed to the conception and design of the study. GS contributed to the conception and design of the study, analyzed the data, and wrote the manuscript. All authors contributed to the article and approved the submitted version.

## Funding

This study was supported by CONACyT grants #_272815 (FOSSIS) and FORDECYT_#302815 (Pronace-Salud). AC-H and SA-C are students of the PhD program, Doctorado en Ciencias Bioquímicas, Universidad Nacional Autónoma de México, and were supported by a fellowship from CONACyT (nos. 549444 and 773064),.

## Conflict of Interest

The authors declare that the research was conducted in the absence of any commercial or financial relationships that could be construed as a potential conflict of interest.

## Publisher’s Note

All claims expressed in this article are solely those of the authors and do not necessarily represent those of their affiliated organizations, or those of the publisher, the editors and the reviewers. Any product that may be evaluated in this article, or claim that may be made by its manufacturer, is not guaranteed or endorsed by the publisher.
