# Supplementary figures and images for: Highly Purified Alloantigen-Specific Tregs From Healthy and Chronic Kidney Disease Patients Can Be Long-Term Expanded, Maintaining a Suppressive Phenotype and Function in the Presence of Inflammatory Cytokines
Source: Front Immunol. 2021 Oct 28;12:686530. doi: 10.3389/fimmu.2021.686530 (PMC8581357; doi:10.3389/fimmu.2021.686530)

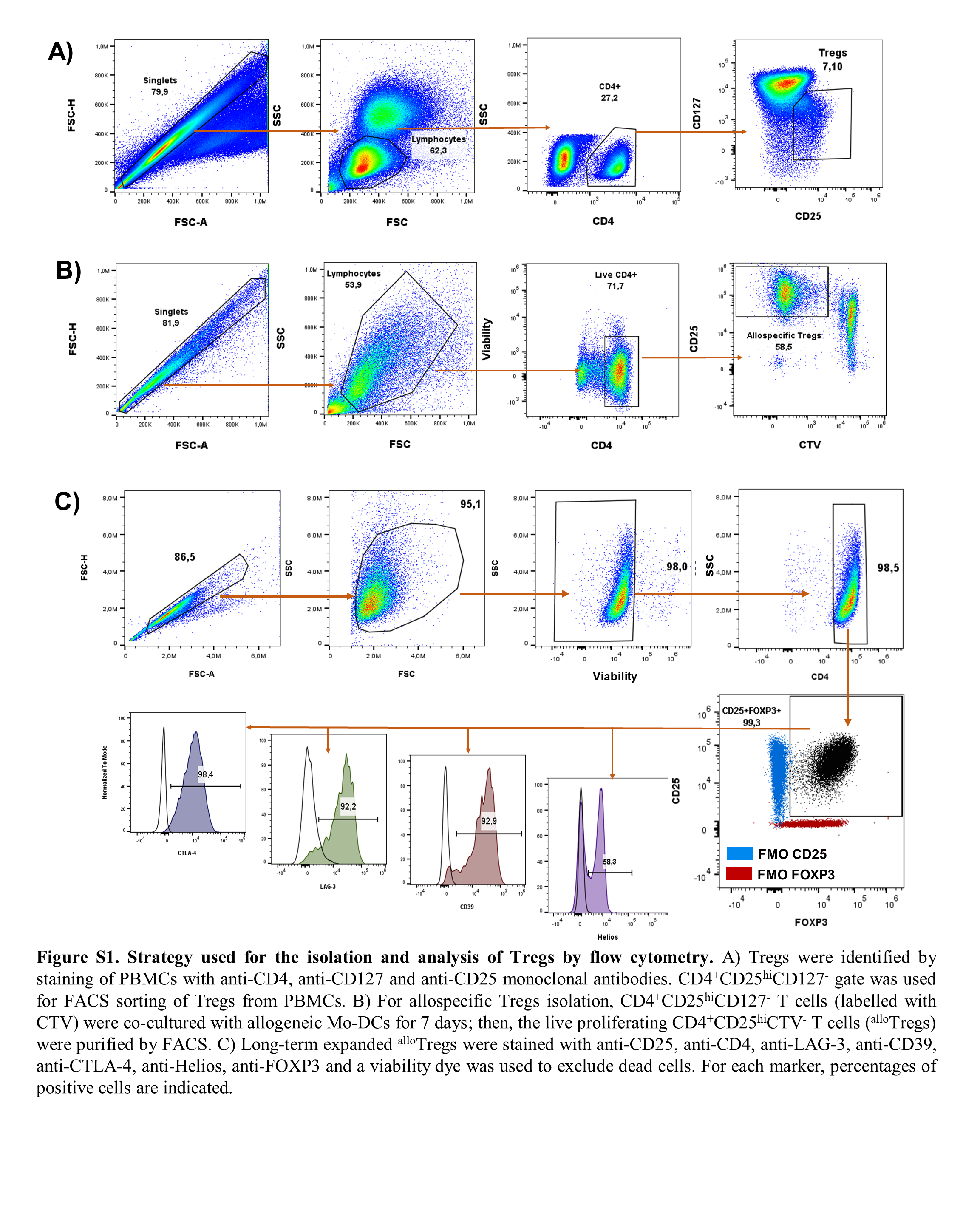

Supplement: Supplementary file 1 [file Image_1.tif]

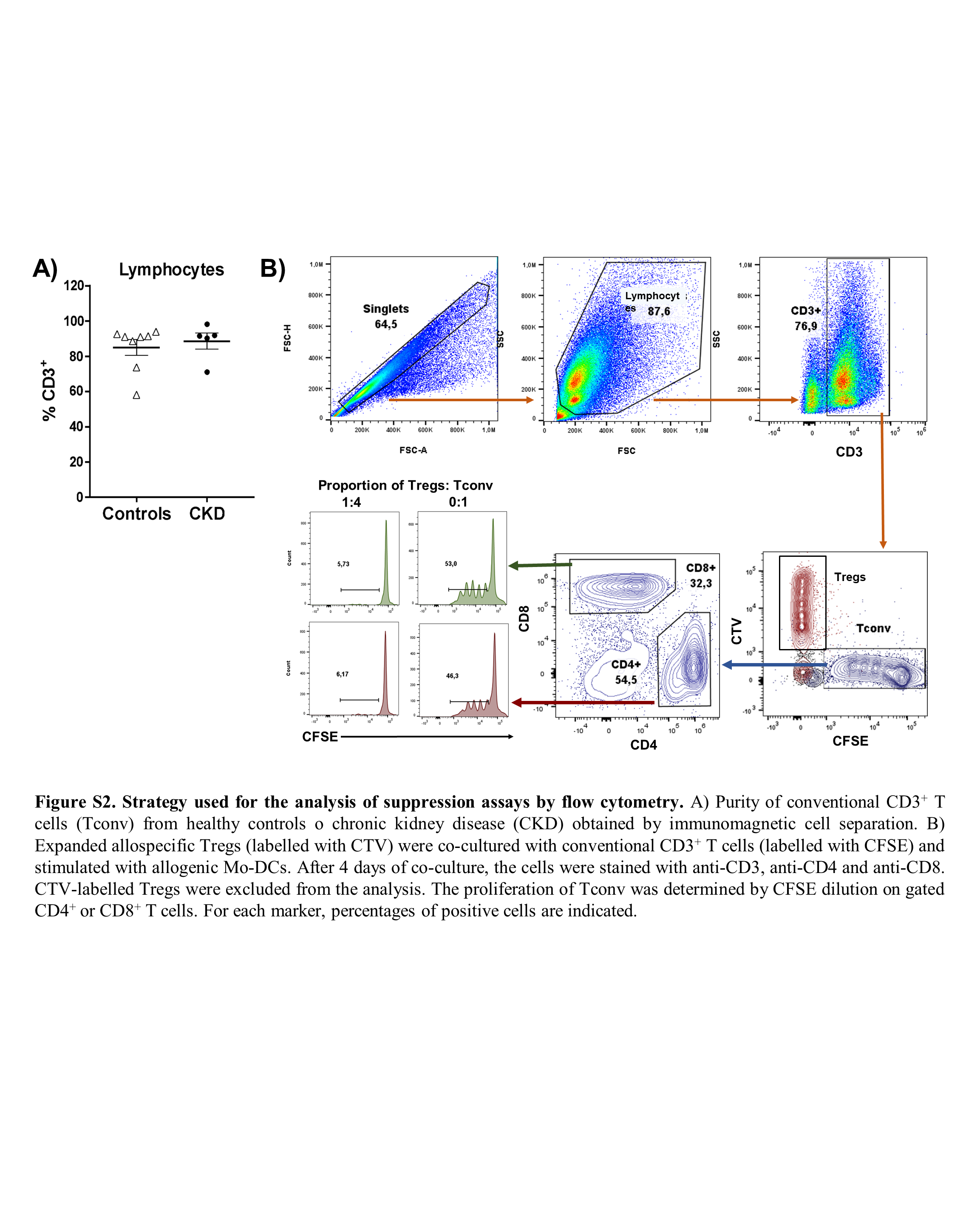

Supplement: Supplementary file 2 [file Image_2.tif]

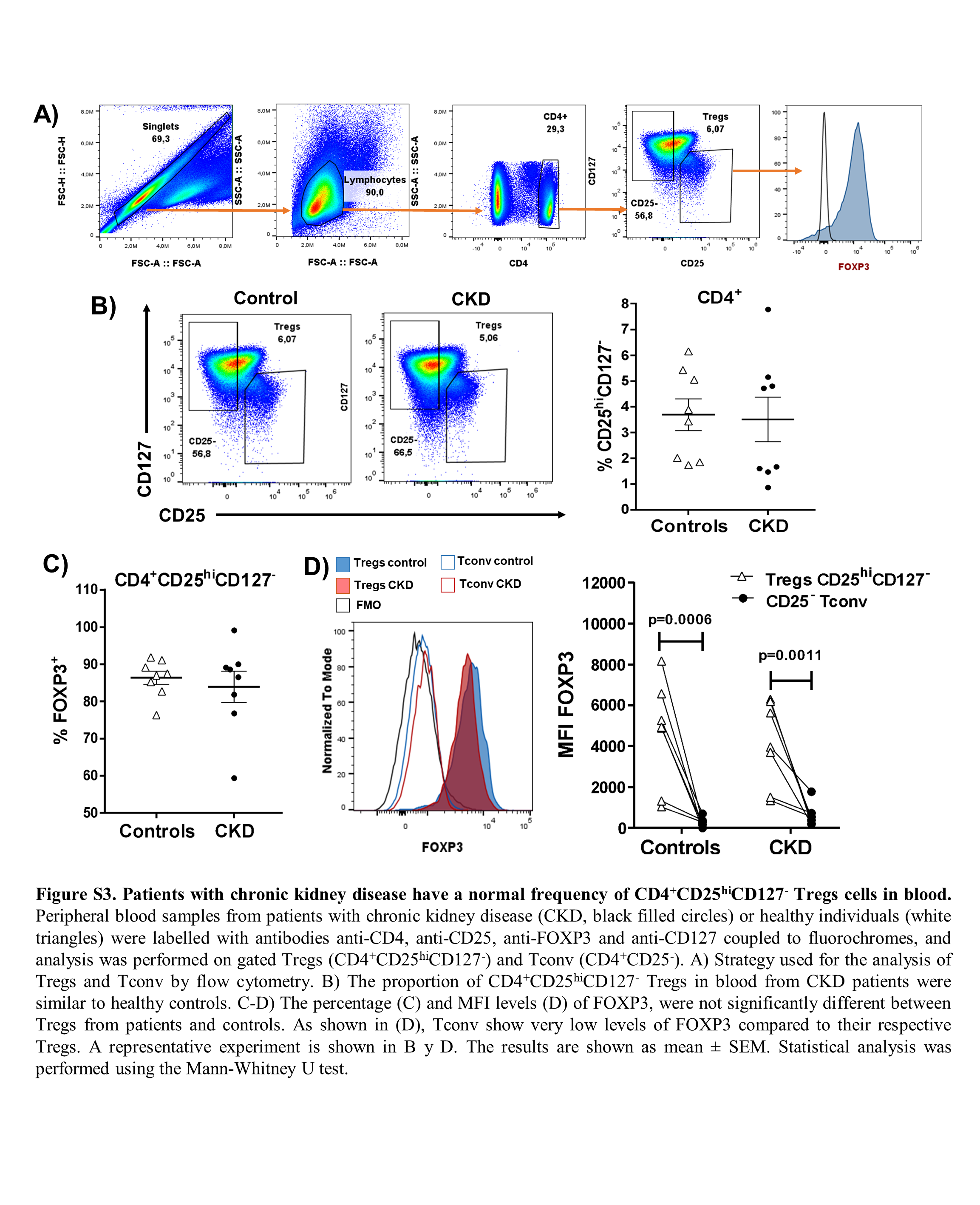

Supplement: Supplementary file 3 [file Image_3.tif]

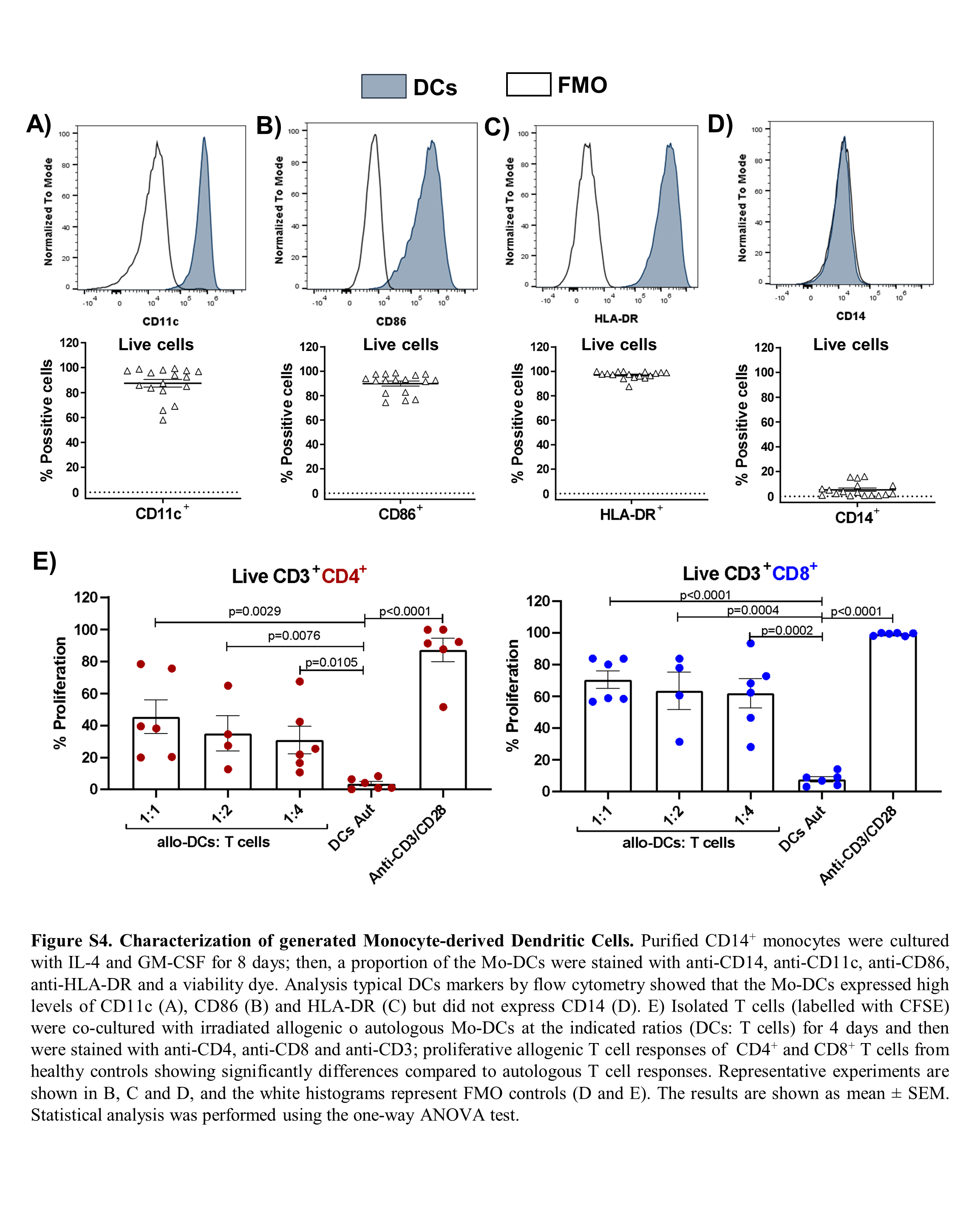

Supplement: Supplementary file 4 [file Image_4.tif]

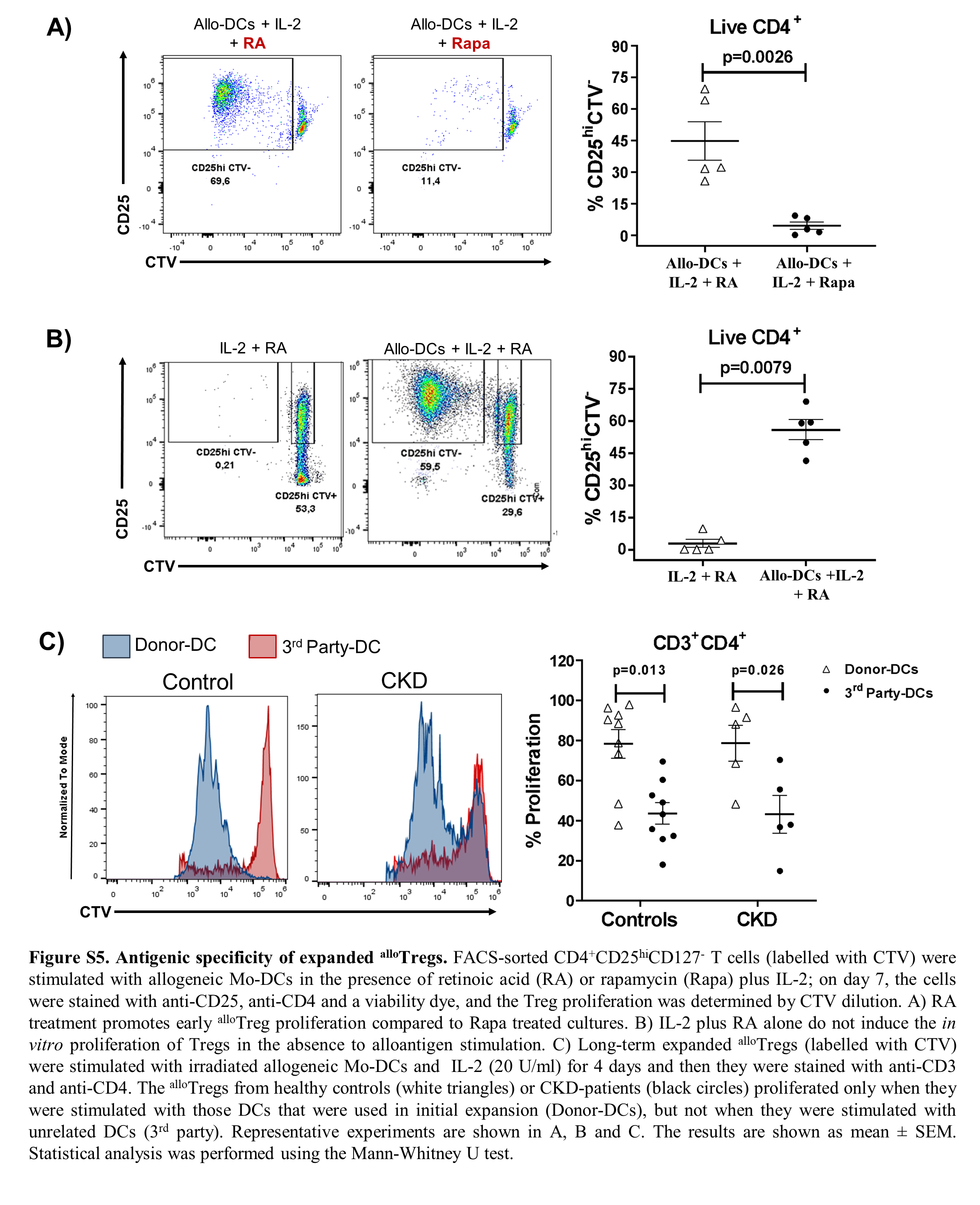

Supplement: Supplementary file 5 [file Image_5.tif]

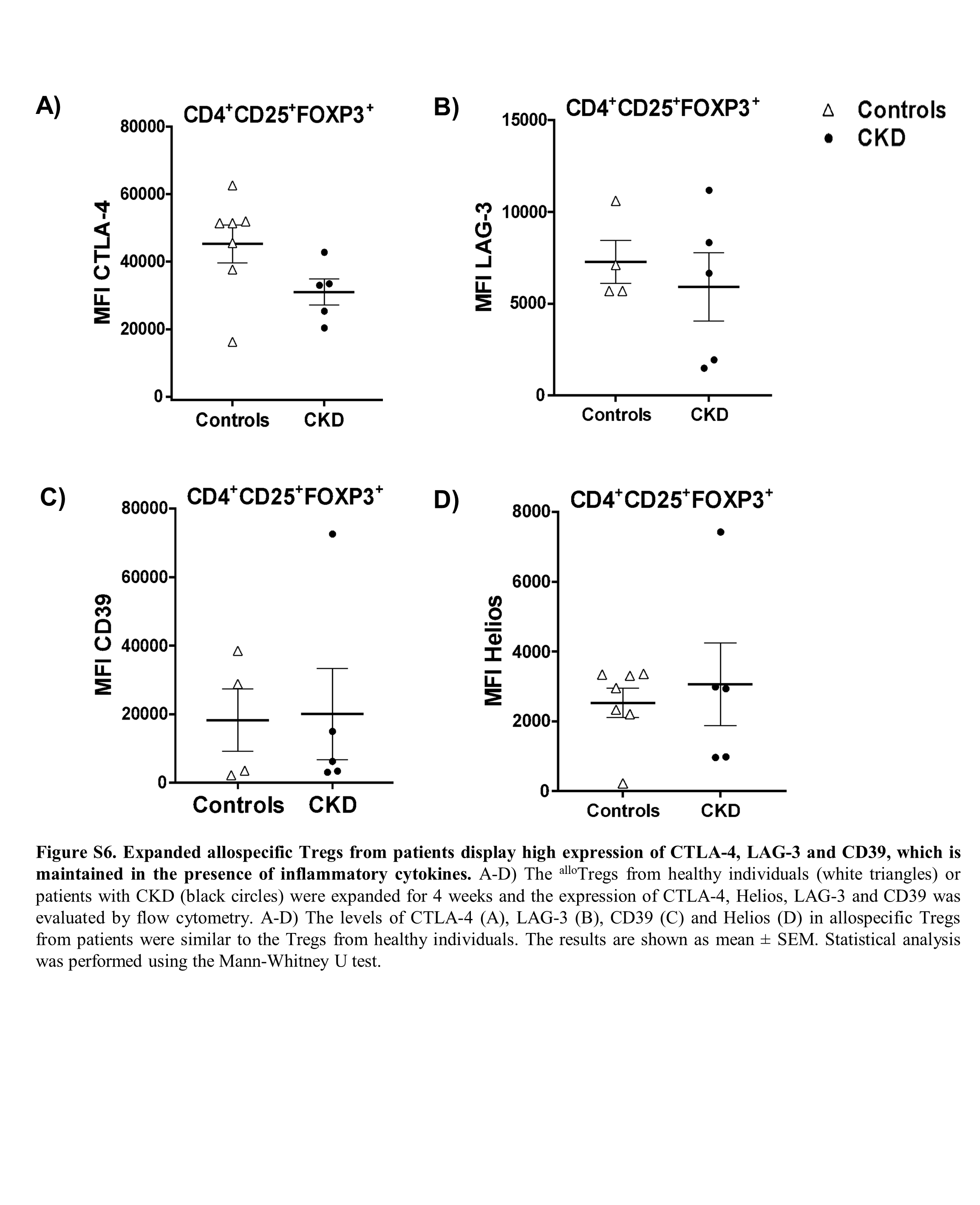

Supplement: Supplementary file 6 [file Image_6.tif]

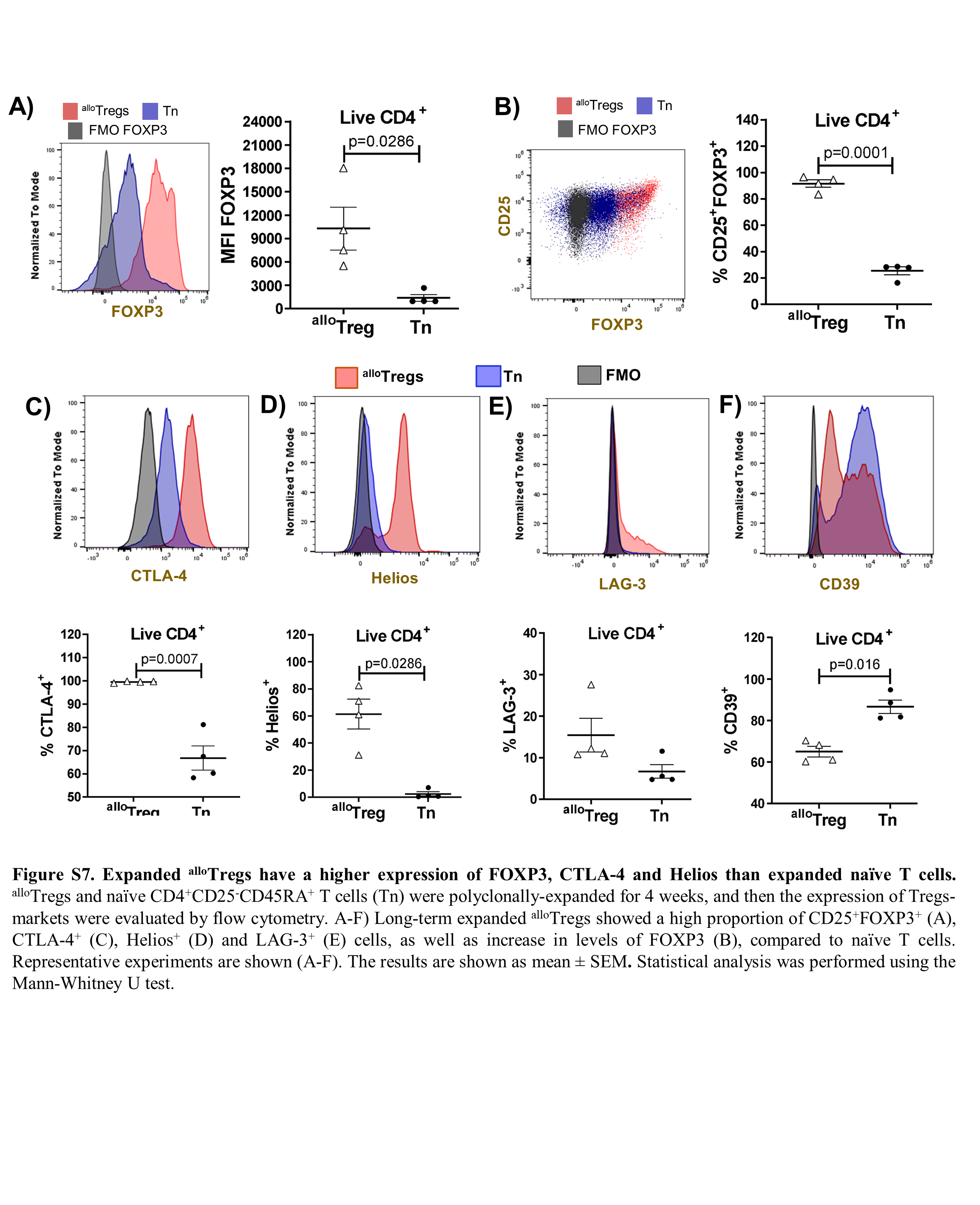

Supplement: Supplementary file 7 [file Image_7.tif]

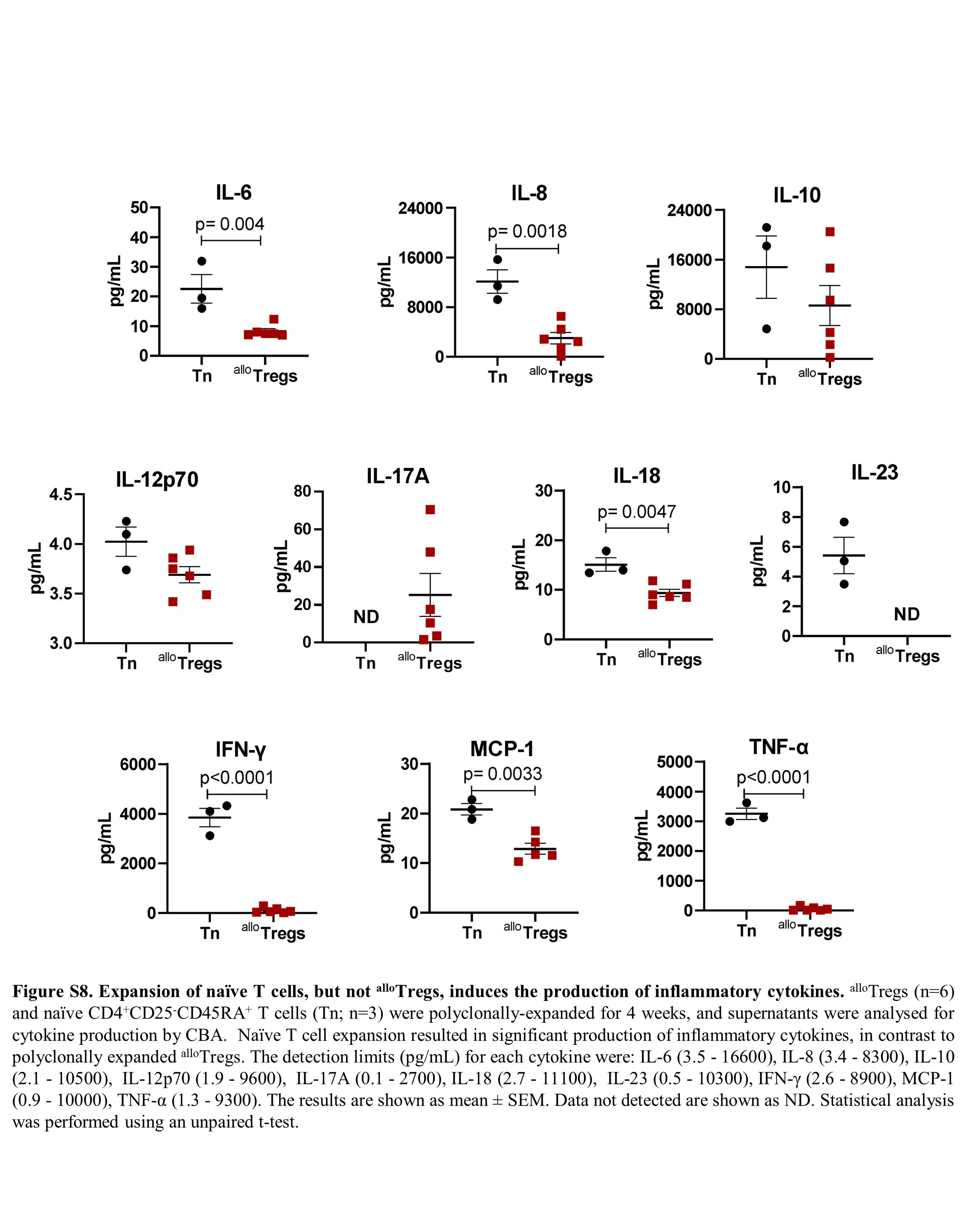

Supplement: Supplementary file 8 [file Image_8.tif]

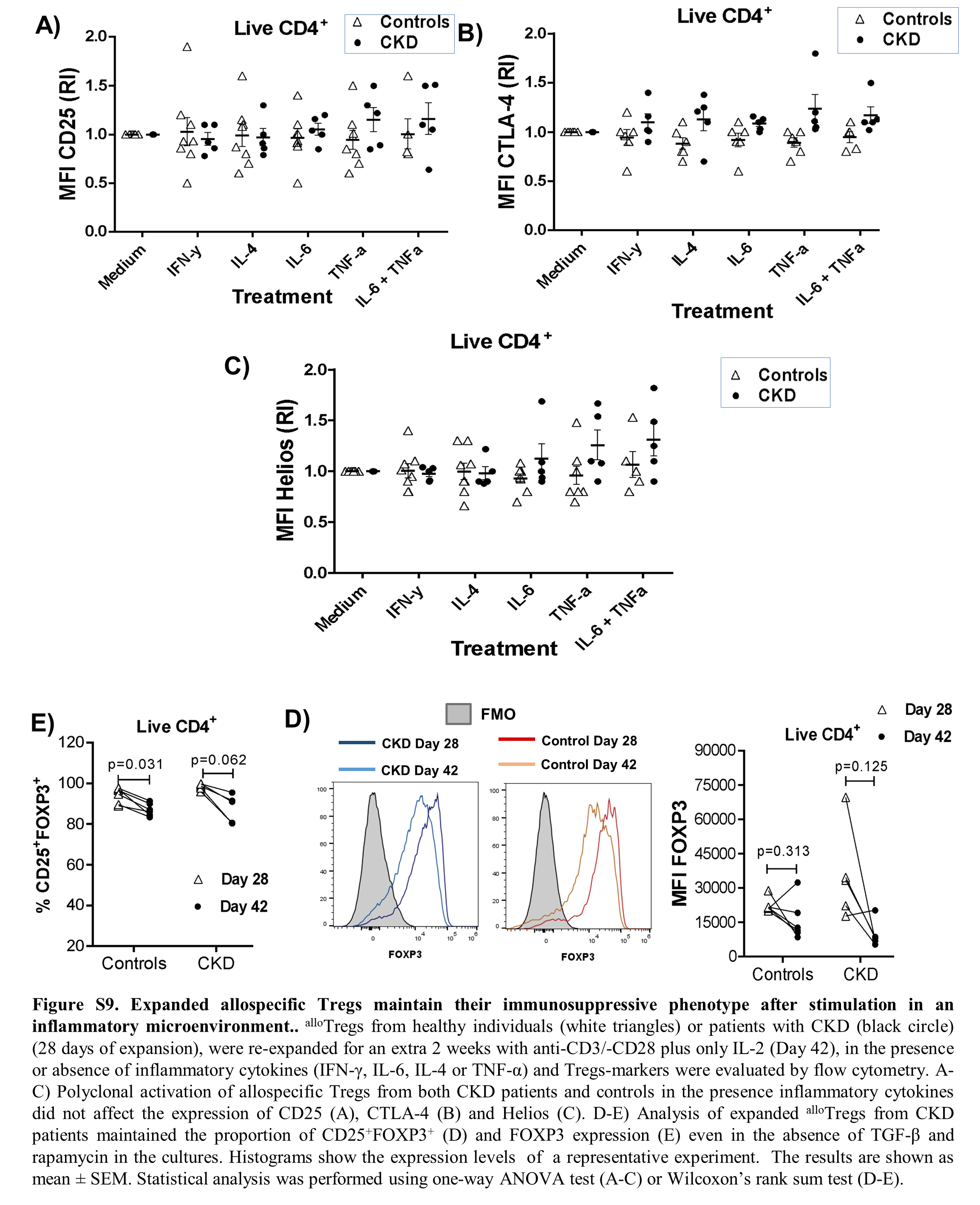

Supplement: Supplementary file 9 [file Image_9.tif]
